# Four Decades of Prophylactic EBV Vaccine Research: A Systematic Review and Historical Perspective

**DOI:** 10.3389/fimmu.2022.867918

**Published:** 2022-04-14

**Authors:** Gabriela M. Escalante, Lorraine Z. Mutsvunguma, Murali Muniraju, Esther Rodriguez, Javier Gordon Ogembo

**Affiliations:** ^1^ Irell & Manella Graduate School of Biological Sciences of City of Hope, Duarte, CA, United States; ^2^ Department of Immuno-Oncology, Beckman Research Institute of City of Hope, Duarte, CA, United States

**Keywords:** Epstein-Barr virus, infectious mononucleosis, cancer, prophylactic vaccine, glycoprotein, neutralizing antibody, herpesvirus, pre-clinical

## Abstract

**Background:**

Epstein-Barr virus (EBV) is the causal agent of infectious mononucleosis and has been associated with various cancers and autoimmune diseases. Despite decades of research efforts to combat this major global health burden, there is no approved prophylactic vaccine against EBV. To facilitate the rational design and assessment of an effective vaccine, we systematically reviewed pre-clinical and clinical prophylactic EBV vaccine studies to determine the antigens, delivery platforms, and animal models used in these studies.

**Methods:**

We searched Cochrane Library, ClinicalTrials.gov, Embase, PubMed, Scopus, Web of Science, WHO’s Global Index Medicus, and Google Scholar from inception to June 20, 2020, for EBV prophylactic vaccine studies focused on humoral immunity.

**Results:**

The search yielded 5,614 unique studies. 36 pre-clinical and 4 clinical studies were included in the analysis after screening against the exclusion criteria. In pre-clinical studies, gp350 was the most commonly used immunogen (33 studies), vaccines were most commonly delivered as monomeric proteins (12 studies), and mice were the most used animal model to test immunogenicity (15 studies). According to an adaptation of the CAMARADES checklist, 4 pre-clinical studies were rated as very high, 5 as high, 13 as moderate quality, 11 as poor, and 3 as very poor. In clinical studies, gp350 was the sole vaccine antigen, delivered in a vaccinia platform (1 study) or as a monomeric protein (3 studies). The present study was registered in PROSPERO (CRD42020198440).

**Conclusions:**

Four major obstacles have prevented the development of an effective prophylactic EBV vaccine: undefined correlates of immune protection, lack of knowledge regarding the ideal EBV antigen(s) for vaccination, lack of an appropriate animal model to test vaccine efficacy, and lack of knowledge regarding the ideal vaccine delivery platform. Our analysis supports a multivalent antigenic approach including two or more of the five main glycoproteins involved in viral entry (gp350, gB, gH/gL, gp42) and a multimeric approach to present these antigens. We anticipate that the application of two underused challenge models, rhesus macaques susceptible to rhesus lymphocryptovirus (an EBV homolog) and common marmosets, will permit the establishment of *in vivo* correlates of immune protection and attainment of more generalizable data.

**Systematic Review Registration:**

https://www.crd.york.ac.uk/prospero/display_record.php?RecordID=198440, identifier PROSPERO I.D. CRD4202019844.

## 1 Introduction

In 2011, the U.S. National Institutes of Health held a meeting on Epstein-Barr virus (EBV) that highlighted the urgent need to develop strategies to prevent EBV infection and EBV-associated diseases (1). Indeed, EBV (also known as human herpesvirus 4) has a global infection rate of more than 90%, and each year, it is associated with ~200,000 new cases of lymphoid and epithelial cancers, resulting in ~145,000 deaths world-wide (1, 2). Moreover, it is the causal agent of infectious mononucleosis (IM), leading to more than 125,000 annual cases of IM in the U.S. alone (3), and is associated with the development of various autoimmune disorders (4–6). Nevertheless, more than a decade later, EBV remains without a clinically approved prophylactic vaccine.

EBV was first discovered by Dr. M.A. Epstein, Dr. B.G. Achong, and Dr. Y.M. Barr in Burkitt lymphoma samples from a Ugandan child in 1964 (7). In 1968, it was identified as the causal agent of IM (8). Two years later, it was further identified as the causal agent of nasopharyngeal carcinoma (9, 10). In 1981, EBV was linked to post-transplant lymphoproliferative disorders in renal transplant patients, an association that is now well-established in other solid-organ transplants and hematopoietic stem cell transplants (11, 12). The virus was subsequently linked to two additional lymphomas, Hodgkin lymphoma in 1987 (13) and T-cell lymphoma in 1988 (14), and was later associated with other lymphoid lymphoproliferative disorders, such as natural killer (NK) cell lymphoma, NK/T-cell lymphoma, and NK-cell leukemia (12, 15). The role of EBV infection in the development of some gastric cancers was suggested in the early 1990s (16, 17), and strengthened in more recent studies, including a meta-analysis (18–23). This year, a 20-year a longitudinal study established EBV as the main causal agent of multiple sclerosis (5), with an additional study identifying the EBV protein EBNA1 as a source of cross-reactive antibodies that also target an adhesion molecule expressed in the central nervous system (6), providing a pathological basis for the role of EBV in multiple sclerosis development.

Motivated by the early association of EBV with several human cancers, Dr. Epstein proposed in 1976 the development of a prophylactic vaccine against EBV as a strategy to prevent EBV infection, to prove that EBV is the causal agent of these cancers, and potentially to reduce the burden of EBV-associated cancers (24). Since then, many prophylactic vaccine candidates have been tested in pre-clinical trials and four Phase I/II clinical trials, but to date, none has moved to a Phase III clinical trial.

**Figure 1:**
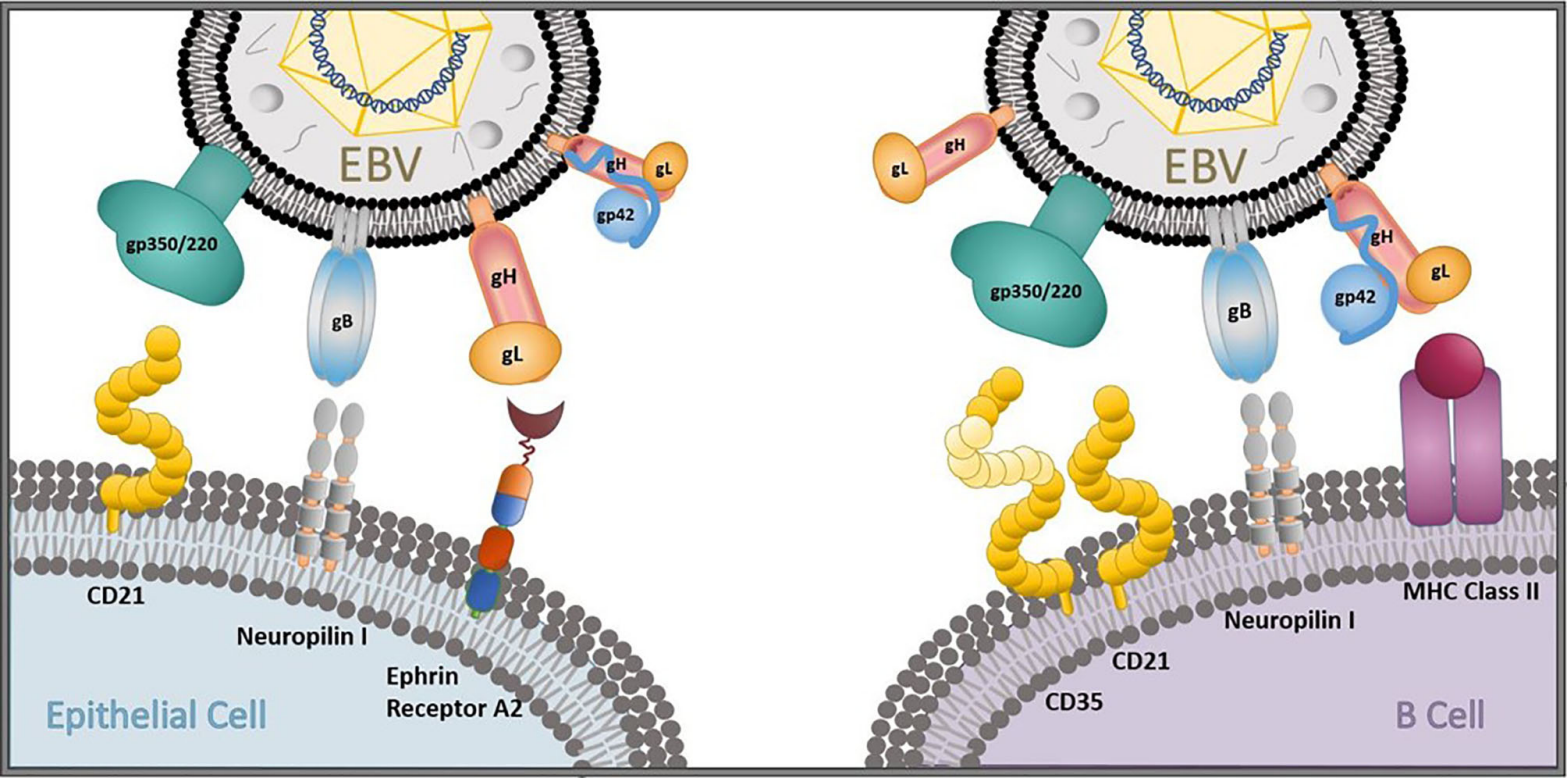
EBV entry and infection of primary target cells. The EBV glycoproteins gp350/220 (gp350), gB, gp42, and the gH/gL complex are important for viral entry into epithelial cells and B cells. gp350 binds to CD21 on epithelial cells and CD35/CD21 on B cells. gB and gH/gL attach to neuropilin 1 and ephrin receptor A2, respectively, on oropharyngeal epithelial cells to gain viral entry, and gB interacts with neuropilin 1 on B cells. gp42 forms a complex with gH/gL that binds to MHC class II to gain viral entry into B cells in collaboration with gB.

Neutralizing antibody (nAb) responses correlate with protection in all licensed antiviral prophylactic vaccines (25), including vaccines against other human herpesviruses, such as varicella-zoster virus and herpes-simplex virus 1 (26). In addition, while cellular immunity plays an essential role in controlling EBV replication and re-activation once primary infection has taken place (27–29), it is humoral immunity against viral entry proteins that can prevent primary infection from taking place. Thus, the majority of prophylactic EBV vaccine efforts to date have focused on generating nAbs that can prevent the initiation of viral entry in multiple permissive cell types *in vitro* (30–32). The attachment protein gp350/220 (gp350, previously known as gp340) and four core fusion glycoproteins—gB, gp42, and the gH/gL complex—are important for EBV entry into both epithelial cells and B cells (**Figure 1**), the main cellular targets of EBV, making these antigens attractive nAb targets for developing an effective prophylactic vaccine (30). Indeed, nAbs against each of these five EBV glycoproteins have been identified, isolated, and fully characterized for their potency in blocking EBV infection *in vitro* and, in some cases, *in vivo* (**Table 1**). Despite this knowledge and discoveries, it is not known which EBV glycoproteins are required to elicit an effective protective response against primary infection, and the correlates of immune protection against primary EBV infection remain undefined. Furthermore, there is no fully validated EBV challenge animal model in which to test vaccine efficacy and explore correlates of immune protection, and there is a lack of knowledge regarding the ideal vaccine delivery platform to present relevant EBV antigens.

**Table 1:** List of EBV glycoprotein-specific monoclonal nAbs.

Year reported	Antibody	Specificity	Species	Reference
1980	72A1	gp350	Murine	(33)
1980	C1	gp350	Murine	(34)
1982	F-2-1 and G-3-1	gp42	Murine	(35)
1988	E1D1	gL	Murine	(36)
2000	CL40 and CL59	gH	Murine	(37)
2018	AMMO1	gH/gL	Human	(38)
2018	AMMO5	gB	Human	(38)
2019	HB5	gp350	Murine	(39)
2020	1D8	gH/gL	Human	(40)

To develop a successful prophylactic vaccine against EBV infection, it is necessary to thoroughly investigate all obstacles faced in previous pre-clinical and clinical EBV vaccine candidate studies and guide effective strategies to circumvent them. In this review, we set out to systematically identify all pre-clinical and clinical studies evaluating antibody-based prophylactic EBV vaccine candidates up to June 20, 2020. Specifically, we sought to determine the type and frequency of EBV antigens used in vaccine candidates, the type and frequency of vaccine platforms used (including routes, doses, adjuvants, and immunization schedules), the type of assays used to measure vaccine efficacy, and in the case of pre-clinical studies, the type and frequency of animal/disease models used to test vaccine immune responses. Herein, we report our findings and identify weaknesses in the design of prior pre-clinical vaccine testing studies, including lack of transparency and completeness in reporting methodology. We also provide recommendations to guide the future rational design and evaluation of prophylactic EBV vaccine candidates that can finally be translated to the clinic to reduce the global health burden of EBV-associated diseases.

## 2 Methods

This systematic review adheres to the Preferred Reporting Items for Systematic Reviews and Meta-Analyses (PRISMA) Statement (**Tables S1**, **S2**) (41). Our protocol was registered with the International Prospective Register of Systematic Reviews (PROSPERO; CRD42020198440) in July 2020.

### 2.1 Search Strategy

We searched the electronic databases of the Cochrane Library, ClinicalTrials.gov, Embase, PubMed, Scopus, Web of Science, the World Health Organization’s Global Index Medicus (WHO-GIM), and Google Scholar for pre-clinical and clinical prophylactic EBV vaccine studies (**Table 2**). The search was performed with a publication date limit of June 20, 2020, and duplicate articles were automatically removed after electronic comparison across databases (EndNote).

**Table 2:** Electronic database search.

Electronic database	Search terms
Cochrane Library	(“epstein barr” OR “ebv” OR “herpesvirus 4”) AND (“vaccin*” OR “immuniz*” OR “immunis*”)
ClinicalTrials.gov	“epstein barr” OR ebv OR “herpesvirus 4” and vaccine
Embase	#1: ‘epstein barr’:ab,ti OR ‘ebv’:ab,ti OR ‘herpesvirus 4’:ab,ti #2: ‘vaccin*’:ab,ti OR ‘immuniz*’:ab,ti OR ‘immunis*’:ab,ti #3: #1 AND #2
PubMed	(“Epstein-Barr Virus Infections”[MeSH] OR “Herpesvirus 4, Human”[MeSH] OR epstein barr[tiab] OR ebv[tiab] OR herpesvirus 4[tiab]) AND (“Vaccines”[MeSH] OR “Vaccination”[MeSH] OR “Immunization”[MeSH] OR vaccin*[tiab] OR immuniz*[tiab] OR immunis*[tiab])
Scopus	(“epstein barr” OR “ebv” OR “herpesvirus 4”) AND (“vaccin*” OR “immuniz*” OR “immunis*”)
Web of Science	#1: TS=(“epstein barr” OR “ebv” OR “herpesvirus 4”) #2: TS=(“vaccin*” OR “immuniz*” OR “immunis*”) #3: #1 AND #2
WHO GIM	(“epstein barr” OR ebv OR “herpesvirus 4”) AND (vaccin* OR immuniz* OR immunis*)
Google Scholar	(“epstein barr” OR ebv OR “herpesvirus 4”)(vaccin* OR immuniz* OR immunis*)

### 2.2 Selection Criteria and Data Extraction

**Figure 2:**
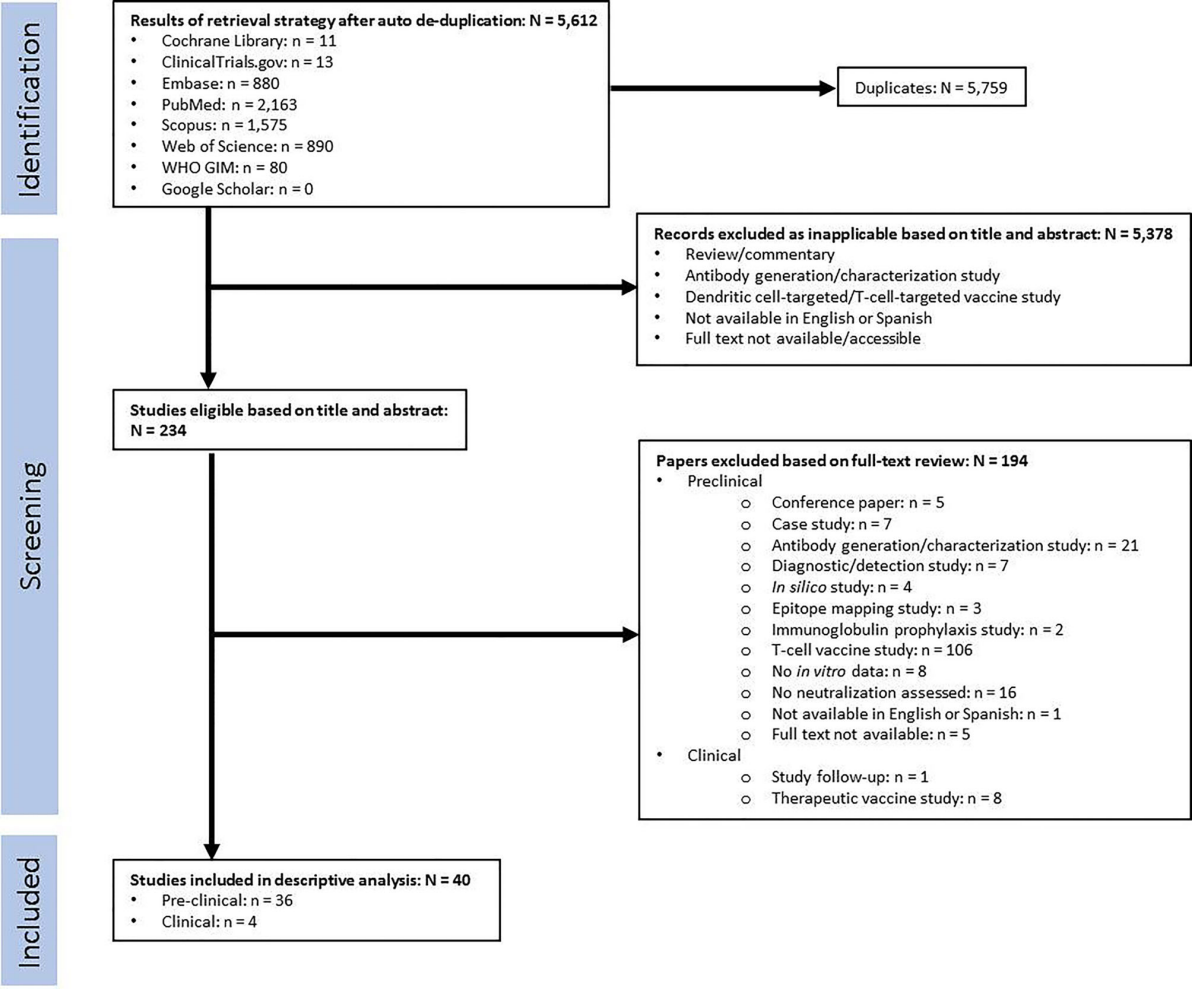
Flow chart for study selection.

Articles were excluded if they were not in English or Spanish and if the full text was not available. Selection based on content was performed in two steps: first based on the title and abstract, then by reviewing the full text. In the first step, articles were excluded based on the nature of the article (review/commentary/antibody development study) and the type of targeted immune response (dendritic cell-targeted/T-cell-targeted vaccines). In the second step, pre-clinical studies were excluded based on the nature of the article (conference paper/case study/antibody generation or characterization study/diagnostic or detection study/*in silico* study/epitope mapping study/immunoglobulin prophylaxis study); the type of targeted immune response (T-cell-targeted vaccines); and lack of data (no *in vitro* data/no neutralization assessed). Clinical studies were excluded based on the nature of the article (study follow-up) or the type of targeted immune response (therapeutic vaccine). Both selection steps were performed by two independent reviewers (JGO and LZM), and any disagreements were resolved by a third reviewer (GME). A flowchart of this process is presented in **Figure 2**.

**Figure 3:**
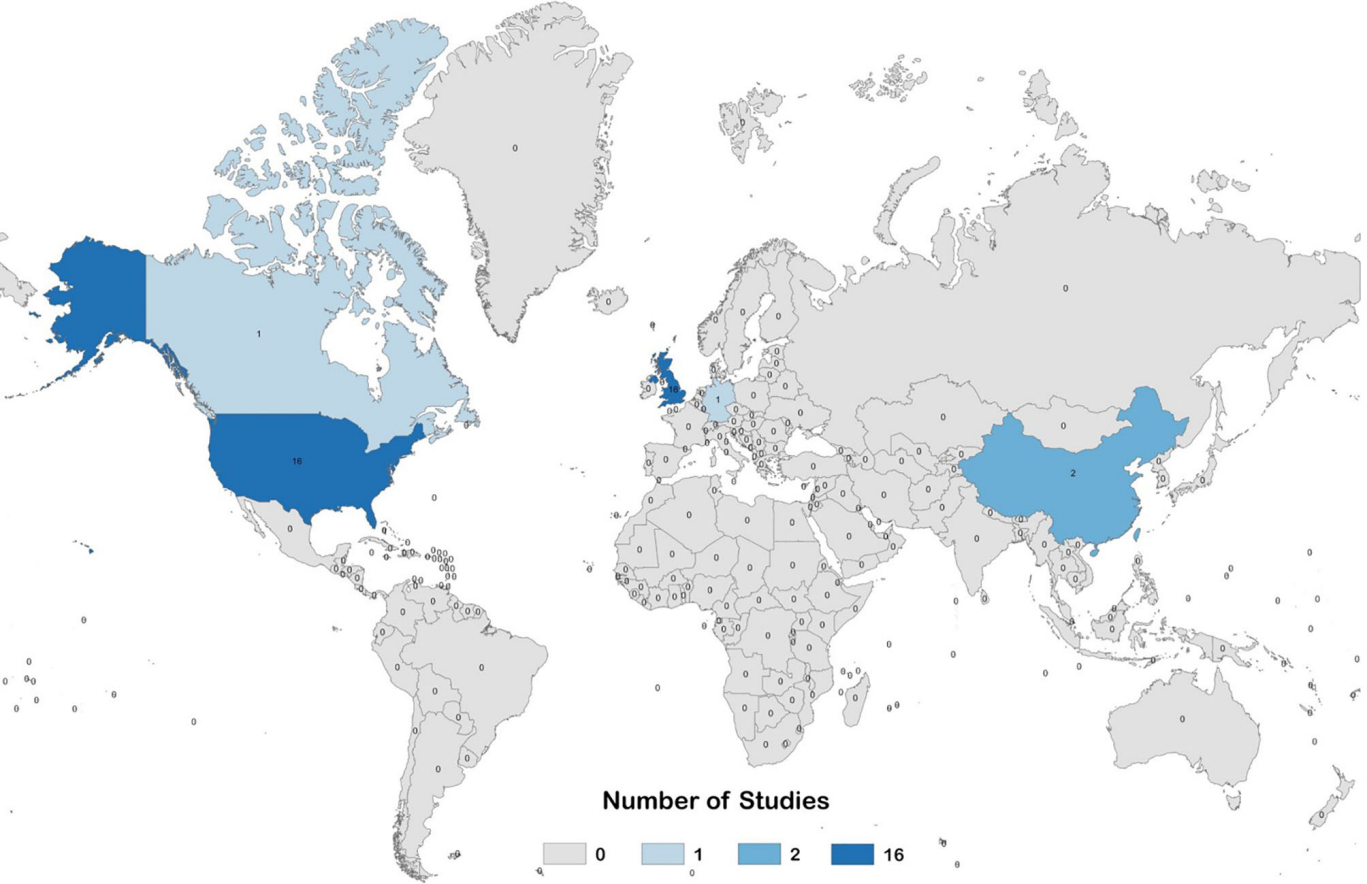
Global heat map describing the number of pre-clinical studies evaluating prophylactic EBV vaccines per country.

**Figure 4:**
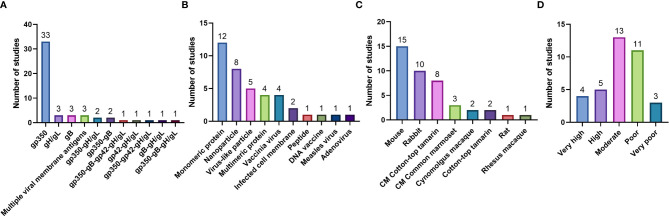
Pre-clinical studies included in the systematic review, enumerated based on **(A)** Immunogen tested; **(B)** Type of vaccine delivery platform used; **(C)** Animal model used to test vaccine immunogenicity and efficacy (CM = challenge model); and **(D)** Quality assessment.

Data extraction was performed by the JGO, GME, LZM, MM and ER. Review of extracted data was performed by JGO and GME, with any disagreements reconciled by LZM. For pre-clinical studies, the following information was extracted: bibliographic information (first author, publication year, title); animal model; characteristics of the population (animal strain, number of animals); characteristics of vaccine (type and source of antigen, dosage, adjuvant, administration route, vaccination schedule); characteristics of control treatments (type of placebo and non-placebo controls, dosage, administration route, vaccination schedule); characteristics of measurement techniques (sample collection schedule, type of antibody detection assay, type of neutralization assay and type of cells used for neutralization assay); and study outcomes. For clinical studies, the following information was extracted: bibliographic information (first author, publication year, title); trial characteristics (country of origin, phase, type, primary endpoint); study population characteristics (age, EBV status, sex, number of participants); characteristics of vaccine and control treatments (type and source of antigen, dosage, adjuvant, administration route, vaccination schedule); characteristics of measurement techniques (sample collection schedule, type of antibody detection assay, type of neutralization assay and type of cells used for neutralization assay, type of EBV diagnostic test); and trial outcomes. Extracted data is presented in **Table 3** for pre-clinical studies, and **Table 4** for clinical studies. For pre-clinical studies, the author list affiliations were individually checked to determine the country of origin for each study, and a global heat-map was prepared displaying the number of studies performed per country (**Figure 3**). From the pre-clinical study extracted data, a list was prepared describing the immunogens tested, the type of vaccine delivery platforms used, and the animal models used to test vaccine immunogenicity and efficacy; the number of studies per variable in each category were graphed and are presented in **Figures 4A–C**.

**Table 3:** Characteristics of the pre-clinical studies included in the systematic review.

First author, year of publication, and title *(glycoprotein[s] targeted)*	Animal model	Vaccine	Study features	Measurement of overall antibody response and efficacy	Study outcomes	Relevance to the field
1. Zhang, 2020 (42) *A novel vaccine candidate based on chimeric virus-like particle displaying multiple conserved epitope peptides induced neutralizing antibodies against EBV infection* *(gp350)*	Female BALB/c mice (n = 5)	*Platform:* Virus-like particle (VLP) *Antigen and dose*: 20µg of hepatitis B virus core antigen (HBc149)-based VLPs expressing three conserved gp350 receptor binding domain peptides. Peptide 1 (P1) (aa 16-29), P2 (aa 142-161) and P3 (aa 282-301) from the gp350 receptor binding domain were fused to HBc149 and expressed in bacteria (BL21). The three peptides were inserted into the multiple insertion region of HBc149 (aa 78-82) in different tandem order combinations, yielding five constructs. The five combinations were: P1-L-P2-L-P3 (3A), where L represents an G4SG4S linker, P1-L-P3-L-P2 (3B), P2-L-P1-L-P3 (3C), P2-L-P3-L-P1 (3D) and P3-L-P2-L-P1 (3E). The five constructs were named 149-3A, 149-3B, 149-3C, 149-3D and 149-3E, respectively *Adjuvant*: Aluminum hydroxide	*Control(s)*: Wild-type HBc149-based VLP (n = 5) or recombinant gp350-ectodomain_123_ protein (1-425aa) (n = 5). *Immunization schedule*: Three subcutaneous injections administered at week (0, 2, 4) *Sample collection*: Week (0, 1, 2, 3, 4, 5, 6, 8, 10 and 14) *Virus challenge*: NA *Study duration*: 14 weeks	*Antibody assay(s)*: ELISA (gp350^1-425^-His) using sera from immunized mice. *In vitro neutralization assay(s)*: Competitive ELISA (gp350^1-425^-His) against anti-gp350 Ab 72A1 using sera from immunized mice; neutralization assay using Akata-EBV-eGFP (from CNE2-EBV cells) in EBV-negative Akata cells, with sera from immunized mice (Week 8 and 10). Neutralization efficacy was reported as % infected cells. *Measurement of in vivo infection:* NA	Sera from mice immunized with 149-3A and 149-3B showed higher anti-gp350 antibody titers and better competition to neutralizing antibodies (nAbs) 72A1 compared to sera from mice immunized with gp350 ectodomain_123_ or other VLPs. At 5-fold and 10-fold dilution, the sera inhibited more than 50% binding of 72A1-HRP. Sera from mice immunized with 149-3A and 149-3B showed stronger neutralizing efficiency compared to the sera raised against soluble gp350 ectodomain_123,_ or other VLPs. At 2-fold, 4-fold, and 8-fold dilutions, the sera raised against 149-3A and 149-3B showed over 50% neutralizing efficiency against EBV infection. The corresponding ID_50_ values are 13.43 and 18.93 for sera from animals immunized by 149-3A and 149-3B, respectively.	This was the first study to incorporate multiple gp350 receptor binding domain epitopes on the surface of chimeric HBc149 VLPs as vaccine candidates. Promising results from this study provide support for a VLP-based vaccine strategy that incorporates multiple known immunogenic and neutralizing epitopes. In addition, the use of different epitope combinations proved very informative, revealing that epitope order in such a platform can have drastic effects on vaccine immunogenicity, which will guide future vaccine design strategies using these types of platforms.
2. Escalante, 2020 (43) *A pentavalent Epstein-Barr virus-like particle vaccine elicits high titers of neutralizing antibodies against Epstein-Barr virus infection in immunized rabbits* *(gp350, gB, gp42, gL, gH in combination)*	Female and male New Zealand white rabbits (n = 6)	*Platform*: VLP *Antigen and dose*: 50µg of Newcastle disease virus (NDV)-based VLP incorporating EBV gp350^1-864^, gB, gp42, gL and gH in a single construct produced in CHO cells *Adjuvant:* 500μg aluminum hydroxide (alum) mixed with 50µg monophosphoryl lipid A from *Salmonella enterica* serotype minnesota Re 595 (MPL)	*Control(s)*: TNE buffer (n = 6), 50µg ultraviolet (UV)-inactivated Akata EBV (n = 6), 25µg soluble gp350 ectodomain^4-863^ (n = 6). *Immunization schedule:* Three subcutaneous injections administered at day 0, 28 and 42 *Sample collection schedule:* Day (-7, 14, 35, 49, 70 and 90) *Virus challenge:* NA *Study duration*: 90 days	*Antibody assay(s)*: ELISA (His-tagged g350, gB, gp42, gH/gL from human embryonic kidney cell line 293 (HEK-293 cells) using sera from immunized rabbits. *In vitro neutralization assay(s)*: Neutralization assay using EBV-Akata-eGFP (from AGS cells) in HEK-293 and Raji cells, with purified immunoglobulins (IgGs) from immunized rabbits at a concentration of 1.56-50μg/ml. Neutralization efficacy was reported as % neutralization and IC_50._ *Measurement of in vivo infection:* NA	Rabbits immunized with VLPs elicited high titers of gp350 and gB antibodies. However, low titers of gp42, and gH/gL antibodies were reported. Despite low gp42, gH/gL titers, purified IgGs from immunized rabbits neutralized virus infection in both epithelial and B cells at comparable levels to IgGs from UV-inactivated Akata EBV-immunized rabbits, and better than IgGs from gp350-immunized rabbits.	This was the first study to selectively combine five EBV glycoproteins in a single prophylactic vaccine candidate. Neutralization results are promising and provide support for a multivalent vaccine approach, but whether these results hold true *in vivo* remains to be tested. In addition, the platform requires optimization to achieve more equal glycoprotein expression to elicit high titers of antibodies against all five selected glycoproteins.
3. Bu, 2019 (44) *Immunization with components of the viral fusion apparatus elicits antibodies that neutralize Epstein-Barr virus in B cells and epithelial cells* (gH/gL, gp42-gH/gL, gp350-gH/gL, gp350-gp42-gH/gL)	(a) Female Balb/c mice (n = 5)	*Platform*: Nanoparticle *Antigen and dose*: 0.5µg of gH/gL or gp42-gH/gL *H. pylori* ferritin-based nanoparticles produced in Expi293F cells *Adjuvant*: Sigma Adjuvant System (50%v/v)	*Controls*: 0.5µg of soluble gH/gL (n = 5) or gp42-gH/gL (n = 5). No negative control reported. *Immunization schedule*: Three intramuscular injections at week (0, 3, 14) *Sample collection*: Week (2, 5, 13, 16, 20, 24, 28) *Virus challeng*e: NA *Study duration*: 28 weeks	*Antibody assay(s)*: Luciferase immunoprecipitation system (LIPS) on HEK-293 cells expressing gp42, and gH/gL using sera from immunized mice. *In vitro neutralization assay(s)*: Neutralization assay using EBV-Akata-eGFP (from Akata BX1 cells) in SVKCR2, AGS and Raji cells, using sera from immunized mice. Neutralization efficacy was reported as IC_50._ *Measurement of in vivo infection:* NA	The gH/gL and gp42-gH/gL ferritin-based nanoparticles elicited antibodies in immunized mice that neutralized B-cell and epithelial cell infection better than antibodies from mice immunized with the soluble version of the proteins. Antibodies elicited by gp42-gH/gL ferritin-based nanoparticles neutralized B-cell infection better than gH/gL ferritin-based nanoparticles, but the addition of gp42 had no effect on epithelial cell neutralization.	This was the first study to report that polyclonal antibodies against the gH/gL and gp42-gH/gL complexes are important components of the EBV neutralizing response in naturally infected individuals, which led to this being the first study to selectively use gp42-gH/gL as a complex for immunization. The results further emphasize the relevance of the three proteins as vaccine targets, as well as the importance of using structurally presented antigens in their native forms over monomeric soluble antigens. Furthermore, results from the gp350 combination studies further provide support for multivalent and multimeric vaccine approaches. However, the ability of the vaccine in eliciting protective nAbs *in vivo* remains to be tested.
	(b) Female Balb/c mice (n = 5)	*Platform*: Nanoparticle *Antigen and dose*: 0.5µg of gp350 gL *H. pylori* ferritin-based nanoparticle + 0.5µg of gH/gL gL *H. pylori* ferritin-based nanoparticle, or 0.5µg of gp350 gL *H. pylori* ferritin-based nanoparticle + 0.5µg of gp42-gH/gL gL *H. pylori* ferritin-based nanoparticle; produced in Expi293F cells *Adjuvant*: Sigma Adjuvant System (50%v/v)	*Controls:* 0.5µg of gp350 (n = 5), gH/gL (n = 5) or gp42-gH/gL (n = 5) ferritin-based nanoparticles. No negative control reported. *Immunization schedule*: Three intramuscular injections at week (0, 3, 14) *Sample collection*: Week (2, 5, 13, 16, 20, 24, 28) *Virus challeng*e: NA *Study duration*: 28 weeks	*Antibody assay(s)*: LIPS assay in HEK-293 cells expressing g350, gp42, and gH/gL using sera from immunized mice. *In vitro neutralization assay(s)*: Neutralization assay using EBV-Akata-eGFP (from Akata BX1 cells) in SVKCR2, AGS and Raji cells, using sera from immunized mice. Neutralization efficacy was reported as IC_50._ *Measurement of in vivo infection:* NA	The gp350-gH/gL and gp350-gp42-gH/gL nanoparticle combinations resulted in antibodies that markedly enhanced neutralization in epithelial cells when compared to antibodies from mice immunized with gp350 ferritin-based nanoparticles alone. However, there was no significant effect observed in B cell neutralization.	
	(c) Cynomolgus macaques (*Macaca fascicularis*); *sex not reported* (n =5)	*Platform*: Nanoparticle *Antigen and dose*: 50µg gH/gL or gp42-gH/gL gL *H. pylori* ferritin-based nanoparticle produced in Expi293F cells *Adjuvant*: Sigma Adjuvant System (50%v/v)	*Control*s: 50µg soluble gH/gL or gp42-gH/gL (n = 5). No negative control reported. *Immunization schedule*: Three intramuscular injections at week (0, 4, 12) *Sample collection*: Week (0, 6, 8, 14, and 24) *Virus challeng*e: NA *Study duration*: 24 weeks	*Antibody assay(s)*: LIPS assay in HEK-293 cells expressing gp42, and gH/gL using sera from immunized macaques; fusion-inhibitory assay testing the fusion of either CHO-K1 cells expressing gB, and gH/gL or CHO-K1 cells expressing gB, gp42, and gH/gL with HEK-293 and Daudi cells, respectively, using sera from immunized macaques. *In vitro neutralization assay(s)*: Neutralization assay using EBV-Akata-eGFP (from Akata BX1 cells) in SVKCR2, AGS and Raji cells, using sera from immunized macaques. Neutralization efficacy was reported as IC_50._ *Measurement of in vivo infection:* NA	As in the first mouse study, the gH/gL and gp42-gH/gL ferritin-based nanoparticles elicited antibodies in immunized macaques that neutralized B-cell and epithelial cell infection better than antibodies from macaques immunized with the soluble version of the proteins. Antibodies elicited by gp42-gH/gL ferritin-based nanoparticles neutralized B-cell infection better than gH/gL ferritin-based nanoparticles, but the addition of gp42 had no effect on epithelial cell neutralization. These results were further confirmed/replicated in fusion-inhibitory assays.	
4. Zhao, 2018 (45) *Immunization with Fc-based recombinant Epstein–Barr virus gp350 elicits potent neutralizing humoral immune response in a BALB/c mice model* *(gp350)*	(a) BALB/c mice; *sex not reported* (n = 5)	*Platform*: Multimeric protein *Antigen and dose*: 1 or 20µg of soluble dimeric gp350 ectodomain fused with mouse Fc-IgG2a (full-length, gp350-ECD_FL_-FC, or truncated, gp350-ECD_123_-FC), produced in Sf9 insect cells *Adjuvant*: Imject alum	*Controls*: Soluble monomeric truncated gp350 ectodomain (gp350-ECD_123_-6His) (n = 5). No negative control reported. *Immunization schedule*: Intraperitoneal injections at week (0, 3) *Sample collection*: Week (0, 1, 2, 3, 4, 5) *Virus challenge:* NA *Study duration*: 5 weeks	*Antibody assay(s)*: ELISA (His-tagged gp350) to determine both overall antibody titers and Ig subtypes, using sera from immunized mice. *In vitro neutralization assay(s)*: Competitive ELISA (His-tagged gp350) against anti-gp350 nAb 72A1 using sera from immunized mice; surface plasmon resonance (SPR) antibody competition assay using 72A1 against sera antibodies from immunized mice; neutralization assay using Akata-EBV-eGFP (from CNE2-EBV cells) in EBV-negative Akata cells, with sera from immunized mice. Neutralization efficacy was reported as % infection rate. *Measurement of in vivo infection:* NA	Mice immunized with the dimeric proteins in both intraperitoneal and intranasal studies displayed higher levels of anti-gp350 antibody titers than mice immunized with monomeric gp350, as well as IgG1, IgG2a and IgG2b titers. Similar results were obtained in both competition assays and in neutralization assays. In general, antibodies from mice immunized intraperitoneally with the high dose of dimeric proteins performed better in all assays compared to mice immunized intranasally. Overall, the gp350-ECD_123_-Fc vaccine performed best.	This was the first EBV vaccine study to use an Fc domain to multimerize an EBV glycoprotein. A vaccine using this type of platform is clinically relevant as Fc approaches have been validated in previous clinical trials and such a vaccine would thus face less regulatory hurdles to reach the clinic. This study is also one of the few preclinical studies to interrogate the identity of Ig subtypes elicited by an EBV vaccine candidate. Overall, this study also provides support for moving away from the use of monomeric proteins in favor of multimeric proteins or platforms that provide structural protein support.
	(b) BALB/c mice; *sex not reported* (n = 5)	*Platform*: Multimeric protein *Antigen and dose*: 20 µg of soluble dimeric gp350 ectodomain fused with mouse FC-IgG2a (full-length, gp350-ECD_FL_-Fc, or truncated gp350-ECD_123_-Fc), produced in Sf9 insect cells *Adjuvant*: 25µg CpG1826	*Controls:* Soluble monomeric truncated gp350 ectodomain (gp350-ECD_123_-6His) (n = 5) or PBS (n = 5). *Immunization schedule:* Intranasal immunization at week (0,2) *Sample collection:* Week (0, 1, 2, 3, 4, 5) *Virus challenge:* NA *Study duration*: 5 weeks			
5. Perez, 2017 (46) *Novel Epstein-Barr virus-like particles incorporating gH/gL- EBNA1 or gB-LMP2 induce high neutralizing antibody titers and EBV-specific T-cell responses in immunized mice* *(gH/gL, gB, gp350, gp350-gH/gL, gp350-gB, gB-gH/gL, gp350-gB-gH/gL)*	Female BALB/c mice: (n = 5)	*Platform:* VLP *Antigen and dose*: 10µg of individual NDV-based VLPs (gB-LMP2, gH/gL-EBNA1, or gp350/220 VLP) or VLP combinations of 10µg each (gp350/220 +gB-LMP2, gp350/220+gH/gL-EBNA1, gB-LMP2+gH/gL-EBNA1, or gp350/220+gB-LMP2+gH/gL-EBNA1), produced in CHO cells *Adjuvant*: None	*Controls*: 10µg of UV-inactivated EBV (UV-EBV) (n = 5), or TNE buffer (n = 5). *Immunization schedule*: Three intraperitoneal injections at day (0, 29, 54) *Sample collection*: Day (14, 18, 33, 46, 68, 97) *Virus challenge:* NA *Study duration:* 97 days	*Antibody assay(s)*: ELISA (lytically-induced AGS-Akata cell lysate), using sera from immunized mice. *In vitro neutralization assay(s)*: Neutralization assay using EBV-Akata-eGFP (from AGS cells) in HEK-293 and Raji cells, with sera from immunized mice. Neutralization efficacy was reported as % neutralization. *Measurement of in vivo infection:* NA	All vaccine groups, except for negative control, elicited IgGs in immunized mice that recognized cell lysate from lytically induced AGS-Akata cells. However, neutralization experiment data as presented is not interpretable, so it is unclear which immunization group(s) elicited better nAb responses and whether multivalent immunogen groups outperformed single immunogen groups in eliciting nAbs.	The study attempted to explore selective combination of multiple EBV glycoproteins as vaccine immunogens, following the multivalent trend recently started in the previous decade. While the vaccine was no doubt immunogenic, neutralization data as presented is not interpretable, and thus the relevance of this study is unclear.
6. Heeke, 2016 (47) *Identification of GLA/SE as an effective adjuvant for the induction of robust humoral and cell-mediated immune responses to EBV-gp350 in mice and rabbits* *(gp350)*	(a) Female BALB/c mice (n = 3)	*Platform*: Monomeric protein *Antigen and dose*: 5, 10, or 20µg of adjuvanted soluble gp350 produced in CHO cells. *Adjuvant*: 5µg of TLR4 agonist glucopyranosyl lipid A (GLA) in 2% stable emulsion (SE), or 100µg Aluminum hydroxide (Alum)	*Controls*: 5, 10, or 20µg of unadjuvanted soluble gp350 (n = 3), or PBS (n = 3). *Immunization schedule*: Two intramuscular injections at day (0, 14) *Sample collection*: Day (28) *Virus challenge:* NA *Study duration*: 28 days	*Antibody assay(s)*: ELISA (gp350) to determine anti-gp350 IgG subtypes in sera of immunized mice. *In vitro neutralization assay(s)*: Neutralization assay was performed but strategy or types of cells involved were not provided. Neutralization efficacy was reported as log_2_ nAb titers able to reduce 50% infection. *Measurement of in vivo infection:* NA	Mice immunized with gp350 adjuvanted with GLA/SE performed better at all doses than mice immunized with unadjuvanted gp350 or gp350 adjuvanted with Alum in IgG1 and IgG2a ELISAs and neutralization assays. Minimal to no activity was observed in ELISAs and neutralization assays for mice immunized with unadjuvanted gp350. Gp350 adjuvanted with Alum only displayed meaningful activity in IgG1 ELISAs.	The study reports the first use of GLA/SE as an adjuvant to enhance immune response in animals immunized with gp350 monomeric protein to elicit both humoral and cellular responses, tested in both mice and rabbits (*only humoral responses were reported in this table*). The results support GLA/SE as a potent humoral and cellular adjuvant that performs better than Alum, but its utility in the clinic is yet to be explored as the product is not yet fully characterized and licensed for clinical use. This study is one of the few preclinical studies to interrogate the identity of Ig subtypes elicited by an EBV vaccine candidate and highlights the contribution of different adjuvants to vaccine immune response.
	(b) Female C57BL/6 mice (n = 7-10)	*Platform*: Monomeric protein *Antigen and dose*: 5µg of adjuvanted soluble gp350 produced in CHO cells. *Adjuvant*: 5µg of GLA/2%SE (n = 10), 5µg of GLA in aqueous formulation (GLA-AF) (n = 8), or 2% SE (n = 7)	*Controls:* 5µg of unadjuvanted soluble gp350 (n = 3), 5µg of GLA/2%SE (n = 3), 5 µg of GLA-AF(n = 3), 2%SE (n = 3), or PBS (n = 3). *Immunization schedule*: Two intramuscular injections at day (0, 14) *Sample collection*: Day (28) *Virus challenge:* NA *Study duration*: 28 days	(Same as above)	The results revealed that SE significantly contributes to the generation of nAbs, as adjuvanting with GLA-AF alone did not result in high nAb titers. On the other hand, GLA significantly contributes to the generation of IgG2c responses. The contribution of GLA and SE to IgG1 responses remained unclear.	
	(c) Female BALB/c mice (n = 6)	*Platform*: Monomeric protein *Antigen and dose*: 10µg of adjuvanted soluble gp350 produced in CHO cells. *Adjuvant*: 5µg of GLA/2%SE, or 100µg of Alum	*Controls*: 10µg of unadjuvanted soluble gp350 (n = 6), or PBS (n = 6). *Immunization schedule*: Three intramuscular injections at day (0, 14, 28) *Sample collection*: Day (28, 42, 73, 102, 132, 161, 192, 222, 252, 283, 312, 347) *Virus challenge:* NA *Study duration*: 347 days	(Same as above)	IgG1, IgG2a, and neutralizing responses were stably maintained up to 347 days in mice immunized with gp350 adjuvanted with GLA/SE. IgG1 and neutralizing response were similarly maintained in mice immunized with gp350 adjuvanted with Alum, although nAb titers were lower than in GLA/SE adjuvanted mice. Low IgG21 activity was observed and maintained in mice immunized with unadjuvanted gp350, but no IgG2a or neutralizing activity was observed.	
	(d) Female New Zealand white rabbits (n = 3)	*Platform*: Monomeric protein *Antigen and dose*: 50 or 100µg of adjuvanted soluble gp350 produced in CHO cells. *Adjuvant*: 1 or 2.5µg of GLA/2%SE	*Controls:* PBS (n = 3). *Immunization schedule*: Four intramuscular injections at day (0, 21, 42, 63) *Sample collection*: Day (0, 14, 35, 56, 77) *Virus challenge:* NA *Study duration*: 77 days	*Antibody assay(s)*: ELISA (gp350) using sera of immunized animals. *In vitro neutralization assay(s)*: Neutralization assay was performed but strategy or types of cells involved were not provided. Results were reported as log_2_ nAb titers able to reduce 50% infection. *Measurement of in vivo infection:* NA	All dose combinations were shown to be immunogenic in immunized rabbits and resulted in similar levels of overall anti-gp350 IgG titers and neutralizing titers at the end of the study. However, the differences in dosage number and level to the mouse studies makes it difficult to compare both.	
7. Cui, 2016 (48) *Rabbits immunized with Epstein-Barr virus gH/gL or gB recombinant proteins elicit higher serum virus neutralizing activity than gp350* *(gH/gL, gB or gp350)*	Male New Zealand white rabbits (n = 5)	*Platform*: Multimeric protein *Antigen and dose*: 25µg of tetrameric gp350^1–470^, trimeric gH/gL, or trimeric gB, produced in CHO cells. *Adjuvant*: 6.25μg alum mixed with 50μg 12mer phosphorothioate-modified CpG-ODN optimized for rabbits	*Control*s: 25µg monomeric gp350 (n = 5) or gH/gL (n = 5). Adjuvant alone is listed as an additional control, but no data is shown for them. *Immunization schedule*: Three subcutaneous injections at day (0, 21, and 42) *Sample collection*: Day (0, 10, 31, and 52) *Virus challenge:* NA *Study duration*: 52 days	*Antibody assay(s)*: ELISA (gp350, gB, gH/gL) using sera from immunized rabbits. *In vitro neutralization*: Neutralization assay in Raji and human peripheral blood naïve B cells using B95-8/F-eGFP EBV, with sera from immunized rabbits. Neutralization efficacy was reported as EDI_50_. *Measurement of in vivo infection:* NA	All constructs tested elicited glycoprotein-specific IgGs in immunized rabbits, although multimeric versions performed better than their corresponding monomeric versions. Trimeric and monomeric gH/gL, trimeric gB, and tetrameric gp350-induced nAbs that blocked EBV infection in B cells that were >100-fold, 20-fold, 18-fold, and 4-fold higher, respectively, than monomeric gp350. However, both monomeric and trimeric gH/gL, as well as trimeric gB, performed better than either monomeric or tetrameric gp350.	This is the first study to selectively use EBV gH/gL as a vaccine candidate to elicit nAb in immunized animals, and although gB had been tested before (Lockey et al, 2008), this is the first study do produce a trimeric form as a vaccine candidate. The study results support the use of multimeric forms of gH/gL and gB as components of a vaccine to prevent EBV infection of B cells. Although the vaccine candidates are expected to also provide protection against epithelial cell infection due to the role of gH/gL and gB in this process, the ability of the vaccine candidates to do so was not tested, and thus their utility in this context was not clear.
8. Ogembo, 2015 (49) *A chimeric EBV gp350/220-based VLP replicates the virion B-cell attachment mechanism and elicits long-lasting neutralizing antibodies in mice* *(gp350)*	BALB/c Mice; *sex not reported* (n = 5)	*Platform*: VLP *Antigen and dose*: 10μg of NDV-based VLP incorporating EBV gp350/220, produced in CHO cells. *Adjuvant*: None	*Controls:* 10μg of soluble gp350/220 (n = 5), 10μg of UV-inactivated EBV (n = 5), or TNE buffer (n = 5). *Immunization schedule*: Five intraperitoneal injections at day (0, 43, 172, 183, 218) *Sample collection*: Day (0, 14, 28, 43, 56, 70, 84, 154, 186, 197, 228) *Virus challenge:* NA *Study duration*: 228 Days	*Antibody assay(s)*: ELISA (gp350/220) to determine anti-gp350 IgG general titers and subtype titers in sera of immunized animals *In vitro neutralization assay(s)*: Neutralization assay in Raji cells using EBV B95-8-eGFP or Akata-eGFP produced in B95-8 or AGS cells, respectively, with sera from immunized mice. Neutralization efficacy was reported as % infected cells. *Measurement of in vivo infection:* NA	Overall, the gp350/220 VLP was immunogenic, resulting in long-lasting gp350/220-specific IgGs in immunized mice, with a predominant IgG1 response. Sera from immunized mice was able to neutralize EBV infection of Raji cells at various dilutions, at similar levels than sera from mice immunized with soluble gp350/220 protein. However, overall, immunization with UV-EBV resulted in superior responses.	This study provided proof-of-concept for the use of the NDV-LP platform as a potential EBV vaccine platform. The platform proved immunogenic, and the vaccine is produced in CHO cells, which are FDA-approved for clinical production of biologics. However, results support the use of multiple glycoprotein targets rather than gp350/220 alone, given that immunization with UV-EBV resulted in higher titers of nAbs. This study is one of the few to interrogate the identity of Ig subtypes elicited by an EBV vaccine candidate and the first to use inactivated virus as a positive control.
9. Kanekiyo, 2015 (50) *Rational design of an Epstein-Barr virus vaccine targeting the receptor-binding Site* *(gp350)*	(a) Female BALB/c mice (n = 5)	*Platform*: Nanoparticle *Antigen and dose*: 0.5 or 5µg of gp350 D_123_ ectodomain (1-425aa, D_123_)-ferritin (*Helicobacter pylori*-bullfrog hybrid)- or gp350 D_123_-encapsulin (*Termotoga maritima*)-based nanoparticles, purified from FreeStyle 293F or Expi293F cells *Adjuvant*: 50% (V/V) SAS	*Controls*: 0.5 or 5µg of soluble gp350 ectodomain, full length, (n = 5), or soluble gp350 D_123_ (n = 5). Mice immunized with irrelevant nanoparticle (n = 5) were additionally used for the challenge study. *Immunization schedule:* Three intramuscular injections at week (0, 3, 16) *Sample collection*: At Week (2, 5) and approximately every 5 weeks afterwards until Week 30. *Virus challenge:* Surrogate gp350-expressing vaccinia virus intranasal challenge (1x10^6 PFU), two months after last immunization. *Study duration*: ~ 26 weeks	*Antibody assay*(*s*): ELISA (g350), biolayer interferometry (gp350), and LIPS on a gp350-Renilla luciferase fusion protein, using sera from immunized mice; SPR antibody competition assay using 72A1 against sera antibodies from immunized mice. *In vitro neutralization assay(s)*: Neutralization assay in Raji cells using EBV B95-8/F-eGFP produced in HEK-293/2089 cells, with sera from immunized mice. Neutralization efficacy was reported as IC_50._ *Measurement of in vivo infection:* gp350-expressing vaccinia infection was measured by weight loss.	Mice immunized with either nanoparticle resulted in high serum gp350-specific IgG titers and long-lasting titers of nAbs that performed better than serum antibodies from mice immunized with soluble gp350 ectodomains (full-length and D_123_), with activity from the latter practically non-existent. In the surrogate challenge study, only immunization with gp350 D_123_-ferritin nanoparticle provided protection against infection.	This study provided proof-of-concept for the use of self-assembling ferritin and encapsulin-based nanoparticles as EBV vaccine platforms, and as such it is the first study to use rational structural nanoparticle design for EBV vaccine development. The results of this study also provide strong support for the use of vaccine platforms that provide conformational protein support, and against the use of monomeric soluble protein. However, it is difficult to discern the relevance of the challenge model used, since vaccinia should be able to infect mice both in the presence or absence of gp350.
	(b) Cynomolgus macaques (*Macaca fascicularis*); *sex not reported* (n = 4)	*Platform*: Nanoparticle *Antigen and dose*: 25µg of gp350 D_123_-ferritin (*Helicobacter pylori*-bullfrog hybrid)- or gp350 D_123_-encapsulin (*Termotoga maritima*)-based nanoparticles, purified from FreeStyle 293F or Expi293F cells *Adjuvant*: 50% (V/V) SAS	*Controls*: 50µg soluble gp350 ectodomain, full length (n = 4). No negative control reported. *Immunization schedule*: Three intramuscular injections at week (0, 4, 12) *Sample collection*: At week (0, 6, 8, 14) *Virus challenge:* NA *Study duration*: ~16 weeks	*Antibody assay*(*s*): ELISA (g350) using sera from immunized macaques. *In vitro neutralization assay(s)*: Neutralization assay in Raji cells using EBV B95-8/F-eGFP produced in HEK-293/2089 cells, with sera from immunized macaques. Neutralization efficacy was reported as IC_50._ *Measurement of in vivo infection:* NA	Immunization with the nanoparticles resulted in an increase of nAbs, although these performed similarly to antibodies from macaques immunized with gp350 ectodomain (control). However, these macaques had previous infection with an EBV-homologous lymphocryptovirus and cross-reactive nAbs prior to immunization, so the full relevance of these results is unclear.	
10. Servat, 2015 (51) *Identification of the critical attribute(s) of EBV gp350 antigen required for elicitation of a neutralizing antibody response in vivo* *(gp350)*	Rabbits; *strain and sex not reported* (n = 3)	*Platform*: Monomeric protein *Antigen and dose*: 50μg native soluble gp350^1-860^ or 50μg denatured/alkylated gp350, produced in CHO cells. Adjuvant: 200μl TiterMax (CytRx)	*Controls*: None reported. *Immunization schedule*: Three subcutaneous injections at day (0, 28. 63) *Sample collection*: At (Pre-bleed, day 73) *Virus challenge:* NA *Study duration*: 73 days	*Antibody assay(s)*: ELISA (gp350) using sera from immunized animals. *In vitro neutralization assay(s)*: Neutralization assay in Raji cells using Akata GFP-EBV produced in Akata cells, with sera from immunized rabbits. Neutralization efficacy was reported as log_2_ sera dilution factor that resulted in 50% neutralization. *Measurement of in vivo infection:* NA	Immunization with both types of gp350 resulted in the generation of anti-gp350 IgGs that were reactive to both native (glycosylated) and denatured forms of gp350. However, only native gp350 resulted in generation of nAbs. Additionally, although not discussed in this table, native gp350 produced in CHO cells was the only form of gp350 able to bind to 72A1, among native and denatured gp350 produced in CHO cells, and native gp350 produced in *E. coli*. Likewise, native CHO gp350 competed for CD21 binding against a CD21 antibody, as opposed to denatured gp350.	This was the first study to analyze the conformational requirements of gp350 to generating a nAb response. The study demonstrated that production of native gp350 in mammalian cells results in post-translational modifications, particularly glycosylation, that stabilizes the protein in general as well as the conformation of its main neutralizing epitope, promoting immunogenicity. This highlights the importance of vaccine antigen source, placing mammalian cells as the ideal EBV antigen producers, due to the requirement for post-translational modifications that cannot be met in other types of cells.
11. Tanner, 2015 (52) *Peptides designed to spatially depict the Epstein-Barr virus major virion glycoprotein gp350 neutralization epitope elicit antibodies that block virus-neutralizing antibody 72A1 interaction with the native gp350 molecule* *(gp350)*	Female BALB/c mice (n =4)	*Platform*: Peptide Antigen and dose: 100µg keyhole limpet hemocyanin (KLH)-conjugated gp350 peptide 1 (SKAPESTTTSPTLNTTGFADY), peptide 2 (DDRTLQ L-A-QNPVYLIPETVPYIKWDN), or peptide 3 (GSAKPG NGSYF-A-SVKTEMLGNEID) *Adjuvant*: SAS	*Controls*: None reported. *Immunization schedule*: Two intraperitoneal injections at day (0, 21) *Sample collection*: At (Pre-bleed, day 35) *Virus challenge:* NA *Study duration*: ~35 days	*Antibody assay(s)*: ELISA (gp350 peptide 1, 2 or 3; or gp350-expressing CEM cells) using sera from immunized mice. *In vitro neutralization assay(s)*: Competitive ELISA (gp350-expressing CEM cells) against anti-gp350 Ab 72A1 using sera from immunized mice. *Measurement of in vivo infection:* NA	Immunization with all peptides resulted in the generation of antibodies specific to each peptide, although only peptide 2 and 3 resulted in antibodies capable of recognizing native gp350. Competition assays indicated that sera from p2- and p3-immunized mice were capable of inhibiting 72A1 recognition of native gp350 by 38% and 23%, respectively, with an additive reduction of 51% when anti-p2 and -p3 sera was pooled, while anti-p1 sera did not result in any competition.	This study was the first to computationally model the interaction of gp350 with the neutralizing anti-gp350 antibody, 72A1. This led to the theoretical identification of putative critical peptides involved in 72A1 binding, which were validated *in vitro* and then tested in immunization studies. While *in vitro* characterization studies of the peptides (not discussed in this table) provide strong support for these peptides as important neutralizing epitopes, immunization studies are not fully conclusive since there is no full gp350 ectodomain immunization comparator that could discern whether peptide immunization results in similar or better nAb responses than immunization with full protein. Furthermore, the immunoglobulin heavy and light chain sequences of 72A1 antibody used in this study were later confirmed to be incorrect [see (39, 53)], undermining the conclusion derived from the study.
12. Cui, 2013 (54) *A novel tetrameric gp350^1-470^ as a potential Epstein-Barr virus vaccine* *(gp350)*	(a) Female BALB/C mice (n = 5)	*Platform*: Multimeric protein *Antigen and dose*: 1 or 25µg of tetrameric gp350^1-470^ fused to two universal human tetanus toxoid (TT)-specific CD4+ T-cell epitopes (P_2_ and P_30_) produced in CHO cells *Adjuvant*: 13μg of alum +/− 25μg 30mer stimulatory CpG-ODN	*Controls*: 1 or 25µg monomeric gp350^1-470^ (n = 5) *Immunization schedule*: Two intraperitoneal injections at day (0, 21) *Sample collection*: Day (0, 14, 21, 28, 35) *Virus challenge:* NA Study duration: 35 days	*Antibody assay(s)*: ELISA (gp350) to determine overall anti-gp350 IgG titers, as well as titers of IgG subtypes in sera of immunized mice. *In vitro neutralization assay(s)*: Inhibitory gp350-CD21 binding assay in CD21-expressing human erythroleukemia cells, using fluorescently labeled gp350 and sera from immunized mice (*only performed in mice at the 25µg dose adjuvanted with alum alone*). *Measurement of in vivo infection:* NA	In both protein and DNA immunizations, tetrameric gp350 performed significantly better than monomeric gp350 at both doses tested, eliciting higher titers of gp350-specific IgGs than monomeric protein in immunized mice. Likewise, immunization with tetrameric protein resulted in higher levels of nAbs than immunization with monomeric protein.	This is the first EBV vaccine study to use a multimeric protein approach, both in soluble protein and DNA vaccine forms. Results support the use of multimeric protein over monomeric protein, as the tetrameric gp350 vaccines performed better than monomeric gp350 vaccines in all its formats. While the main tetrameric format of the vaccine included two known TT-specific CD4+ T-cell epitopes, immunization with a tetrameric format that omitted the TT epitopes revealed that the epitopes did not have any effect on vaccine humoral immunogenicity; thus suggesting that the enhanced immunogenicity of the tetrameric vaccine as compared to the monomeric vaccine can be attributed to its multimeric form.
	(b) Female BALB/c mice (n = 7)	*Platform:* DNA vaccine *Antigen and dose:* 4μg plasmid DNA encoding tetrameric gp350^1-470^ fused to TT epitopes P_2_ and P_30_ delivered in gold nanoparticles *Adjuvant:* None	*Controls:* 4μg plasmid DNA encoding monomeric gp350^1-470^ delivered in gold nanoparticles (n = 7) *Immunization schedule:* Two epidermal immunizations in the abdominal skin at week (0, 4) *Sample collection:* Week (0, 4, 6) *Virus challenge:* NA *Study duration:* 6 weeks	*Antibody assay(s)*: As above. *In vitro neutralization assay(s)*: NA *Measurement of in vivo infection:* NA		
	(c) Female BALB/C mice (n = 5)	*Platform:* Multimeric protein *Antigen and dose*: 14-day daily TT administration (0.25 or 25μg, adjuvanted with alum) followed by 25μg of tetrameric gp350^1-470^ fused to TT epitopes P_2_ and P_30_, or by 25μg monomeric gp350^1-470^ *Adjuvant:* 13μg of alum	*Controls:* TT-unprimed 25μg of tetrameric gp350^1-470^ fused to TT epitopes P_2_ and P_30_ (n = 5) or monomeric gp350^1-470^ (n = 5). *Immunization schedule*: Two intraperitoneal injections at day (0, 14) *Sample collection*: Day (0, 7, 14, 21) *Virus challenge:* NA *Study duration:* 21 days	*Antibody assay(s)*: ELISA (gp350) using sera from immunized mice. *In vitro neutralization assay(s)*: NA *Measurement of in vivo infection:* NA	While TT priming did not affect the immunogenicity of monomeric gp350 at both doses tested, it did result in a significant reduction in anti-gp350 IgGs in mice immunized with tetrameric gp350 at the 25μg TT dose, effectively eliminating the immunogenic advantage of the tetrameric gp350 as compared to the monomeric gp350.	
	(d) Female BALB/C mice (n = 5)	*Platform:* Multimeric protein *Antigen and dose:* 1 or 25µg of tetrameric gp350^1-470^ without TT epitopes *Adjuvant:* 13μg of alum	*Controls:* 1 or 25µg monomeric gp350^1-470^ (n = 5), or tetrameric gp350^1-470^ fused to TT epitopes P_2_ and P_30_ (n = 5). *Immunization schedule*: Two intraperitoneal injections at day (0, 21) *Sample collection*: At day (0, 14, 21, 28, 35) *Virus challenge:* NA *Study duration*: 35 days	*Antibody assay(s)*: ELISA (gp350) using sera from immunized mice. *In vitro neutralization assay(s)*: Inhibitory gp350-CD21 binding assay in CD21-expressing human erythroleukemia cells, using fluorescently labeled gp350 and sera from immunized mice (*only performed in mice at the 25µg dose*). *Measurement of in vivo infection:* NA	Removal of TT epitopes from the tetrameric gp350 did not affect the ability of the vaccine to elicit anti-gp350 IgGs, for both protein and DNA vaccines. Similarly, removal of TT epitopes did not affect the ability of tetrameric gp350 to elicit nAbs.	
	(e) Female BALB/c mice (n = 7)	*Platform:* DNA vaccine *Antigen and dose:* 4μg plasmid DNA encoding tetrameric gp350^1-470^ without TT epitopes delivered in gold nanoparticles *Adjuvant:* None	*Controls:* 4μg plasmid DNA encoding tetrameric gp350^1-470^ fused to TT epitopes P_2_ and P_30_ (n = 7), or plasmid DNA encoding monomeric gp350^1-470^ (n = 7), delivered in gold nanoparticles. *Immunization schedule:* Two epidermal immunizations in the abdominal skin at week (0, 4) *Sample collection:* Day (0, ~35, ~50) *Virus challenge: NA* *Study duration:* ~50 days	*Antibody assay(s)*: ELISA (gp350) using sera from immunized mice. *In vitro neutralization assay(s)*: NA *Measurement of in vivo infection:* NA		
13. Mok, 2012 (55) *Evaluation of measles vaccine virus as a vector to deliver respiratory syncytial virus fusion protein or Epstein-Barr virus glycoprotein gp350* *(gp350)*	(a) Cotton Rats (*Sigmodon* spp); *sex not reported* (n = 4)	*Platform*: Measles vaccine virus (Edmonston-Zagreb strain) *Antigen and dose*: 10^5^ PFU of live-attenuated recombinant measles vaccine virus (MV), expressing full-length gp350 ectodomain^1-861^ or truncated gp350 ectodomain (gp350tr^1-470^ produced in HEp-2 cells. *Adjuvant*: None	*Controls*: PBS (n = 3), or 10^5^ PFU of MV not expressing any antigens (rEZ; n = 5). *Immunization schedule*: Two intramuscular injections at week (0, 4) *Sample collection*: Week (4, 6) *Virus challenge:* NA *Study duration*: ~56 days	*Antibody assay(s)*: ELISA (gp350) using sera from immunized rats. *In vitro neutralization assay(s)*: Neutralization assay in Raji cells using B95-8 GFP-EBV produced in B95-8 cells, with sera from immunized rats. Neutralization efficacy was reported as log_2_ sera reciprocal dilution that resulted in 50% neutralization. *Measurement of in vivo infection:* NA	Immunization of rats with MV expressing either form of gp350 elicited gp350-specific IgGs, albeit at low titers, although MV expressing full-length gp350 ectodomain resulted in higher titers than MV expressing gp350tr. However, no EBV nAbs were detected in the sera of immunized rats in either group. In rhesus macaques, the MV-gp350 vaccine did not yield any detectable anti-gp350 IgGs, or EBV nAbs. While not discussed here, T cell responses elicited by the gp350 vaccines were also analyzed, but in both rats and rhesus macaques the cellular responses were low.	The study indicates that the use of measles vaccine virus as a vector to deliver gp350 is not optimal in eliciting nAbs in either cotton rats or rhesus macaques, despite gp350 antibody being detected in immunized cotton rats at low levels. Since increased immunogenicity was observed in a respiratory syncytial virus MV vaccine also tested in the study, and given the differences in immunogenicity observed between rat and NHP immunizations, the study demonstrates that immunogenicity of foreign proteins expressed by measles virus as a vaccine platform is dependent on the nature of the insert and the animal models used for vaccine evaluation. Importantly, the measles vector was shown not to be ideal as a gp350 vaccine platform.
	(b) NHP, Rhesus macaque (*Macaca mulatta*); *sex not reported* (n = 4 measles virus seropositive; n = 4 measles virus naïve)	*Platform*: Measles vaccine virus (Edmonston-Zagreb strain) *Antigen and dose*: 10^5^ PFU of MV expressing full-length gp350 ectodomain^1-861^ produced in HEp-2 cells. *Adjuvant*: None	*Controls*: None reported. *Immunization schedule*: Two intramuscular injections at week (0, 4) *Sample collection*: At week (4, 6) *Virus challenge:* NA *Study duration*: ~56 days	*Antibody assay(s)*: As above. *In vitro neutralization assay(s)*: As above. *Measurement of in vivo infection:* NA		
14. Ruiss, 2011 (56) *A virus-like particle-based Epstein-Barr virus vaccine* *(multiple antigens)*	BALB/c mice; *sex not reported* (n = 4)	*Platform*: VLP *Antigen and dose*: 10μg of exosomal vesicles derived from lytically indued EBV packaging HEK-293 cells (VLPs) that contain various EBV proteins and RNAs, but are devoid of viral DNA *Adjuvant*: None	*Controls*: 10μg exosomes from non-EBV-packaging HEK-293 cells (n = 2). *Immunization schedule*: Two intraperitoneal injections at day (0, 14) *Sample collection*: Week (0, 6) *Virus challenge:* NA *Study duration*: 6 weeks	*Antibody assay(s)*: ELISA, using sera from immunized animals, against cell lysates of HEK-293 cells expressing the following EBV antigens: EBNA1, BFRF3, BMRF1, BKRF4, BVRF1, BDLF3, BZLF2 (gp42), BXLF2 (gH), BNRF1, BALF4 (gB), BZLF1, BLLF1 (gp350); cytomegalovirus pp65 HEK-293 cell lysate was used as a negative control. *In vitro neutralization assay(s)*: Neutralization assay in human primary B cells using GFP-EBV 2089 produced in HEK- 293/2089 cells, with sera from immunized mice. Neutralization efficacy was reported as % infected cells. *Measurement of in vivo infection:* NA	Immunization of mice with the VLPs elicited IgGs specific to each of the antigens tested in ELISA. Furthermore, immunization with VLPs resulted in generation of EBV nAbs that inhibited infection at a similar rate to 72A1 used at 20-40µg/ml).	The study generated an EBV-based VLP that shares the general morphology of the EBV virion, incorporates various viral proteins and RNAs, and, in theory, does not contain any EBV DNA. The platform proved promising in its immunogenicity both in cellular (not discussed here) and humoral responses. However, the feasibility of producing such a vaccine in a large scale, as recognized by the authors, is a challenge, and its production in human cells could face regulatory hurdles. Thus, an alternative production platform might be required for such an approach to reach the clinic.
15. Lockey, 2008 (57) *Epstein-Barr virus vaccine development: a lytic and latent protein cocktail* (gp350, gB*, gp350-gB*)	C57BL/6 mice; *sex not reported* (n =3 or 6)	*Platform*: Recombinant vaccinia virus (VV) vector *Antigen and dose*: 10^7^ PFU recombinant VV produced in MC57G cells expressing individual EBV proteins (n = 3 each): gp350, gp110 (gB), EBNA2, or EBNA2C; or 10^7^ PFU of a combination of all four VVs mixed in equal ratios (n = 6). *Adjuvant*: None	*Controls*: VV expressing the hemagglutinin neuraminidase-of human parainfluenza virus type 1 (VV-hPIV-HN) (n = 3), or no injection (n = 1). *Immunization schedule*: Single intraperitoneal injection at week (0) *Sample collection*: Week (2, 4, 8) *Virus challenge:* NA *Study duration*: 8 weeks	*Antibody assay(s)*: Immunoprecipitation assays against each antigen using sera from immunized mice; immunofluorescence (IFA) assays against B95-8 cells using sera from mice immunized with either VV-gp350, VV-mix, or VV-hPIV-HN. *In vitro neutralization assay(s)*: Neutralization assay (transformation inhibition) in human PBMCs using B95-8 supernatants, with sera from mice immunized with either VV-gp350 or VV-mix. Neutralization efficacy was reported as the highest serum dilution to inhibit transformation in ≥ 3 of 5 test wells, and in ≥ 1 of two independent assays. *Measurement of in vivo infection:* NA	Both individual antigen immunizations as well as the cocktail immunization successfully generated antibodies against each of the antigens tested, with comparable responses between individual antigens and the cocktail. While immunization of mice with VV-gp350 and VV-mix resulted in nAbs (other groups were not tested), titers were low when compared to those found in human sera from naturally infected individuals, which was used as a positive control.	This study proposes the use of a multivalent antigen cocktail that includes both lytic and latent EBV antigens as a vaccine, delivered as a viral vaccine vector. While the study demonstrated the cocktail approach to be immunogenic, resulting in both cellular (not discussed here) and humoral responses, whether this particular cocktail can prevent or treat infection *in vivo* remains unclear. The lack of ELISA data for each of the vaccine antigens also complicates assessment of the antibody response, and the low nAb titers generated by the vaccine suggests additional antigens might be needed to mount a more robust nAb response.
16. Wilson, 1999 (58) *The major Epstein-Barr virus (EBV) envelope glycoprotein gp340 when incorporated into Iscoms primes cytotoxic T-cell responses directed against EBV lymphoblastoid cell lines.* *(gp350)*	NHP, Cottontop tamarins (*Saguinus oedipus oedipus*); *sex not reported* (n =3)	*Platform*: Nanoparticle *Antigen and dose*: 30μg of gp340 produced in murine C-127 cells that were incorporated into immune-stimulating complexes (ISCOMs) *Adjuvant*: ISCOM	Control: Treatment not stated (n = 2) *Immunization schedule*: Three intramuscular injections at week (0, 3, 6). *Sample collection*: Week (7) *Virus challenge:* NA Study duration: ~7 weeks	*Antibody assay(s)*: ELISA (gp340) using sera from immunized tamarins. *In vitro neutralization assay(s)*: Neutralization assay in human fetal cord blood lymphocytes using B95-8 EBV, with sera from immunized tamarins; competitive ELISA (gp340) against anti-gp340 Ab 72A1 using sera from immunized tamarins. Neutralization efficacy was reported as EBV titer, where a reduction of virus titer greater than one log was considered neutralizing. *Measurement of in vivo infection:* NA	Immunization with the ISCOM-adjuvanted protein resulted in the generation of anti-gp340 IgGs. However, the neutralizing activity of the generated antibodies was very low when compared to sera antibodies from EBV-seropositive humans, attributed to low levels of antibody recognizing the major neutralizing epitope of gp340.	This study was the first to incorporate recombinant gp340 ectodomain produced in cell culture into nanoparticles/ISCOMs, a much more feasible approach than using gp350 isolated from EBV-infected cells, as previous gp340-ISCOM studies. Although cellular responses were very promising (not discussed here), humoral responses were suboptimal, perhaps due to gp340 denaturation occurring during chemical coupling to ISCOMs.
17. Jackman, 1999 (59) *Expression of Epstein Barr virus gp350 as a single chain glycoprotein for an EBV subunit vaccine* *(gp350)*	Rabbits; *strain and sex not reported* (n = 3)	*Platform*: Monomeric protein *Antigen and dose*: 50μg (for Freund’s adjuvant) or 100μg (for Alum) of recombinant gp350 non-splicing variant protein produced in CHO cells *Adjuvant*: Freund’s adjuvant or Alum	Controls: None reported. *Immunization schedule*: Three injections at day (0, 21, 42); no injection route stated. *Sample collection*: At (Pre-bleed, day 31, day 52) *Virus challenge:* NA *Study duration*: 52 days	*Antibody assay(s)*: ELISA (non-splicing gp350 variant) using sera from immunized rabbits. *In vitro neutralization assay(s)*: Neutralization assay in purified human B-lymphocytes, using unknown strain of EBV, with sera from immunized rabbits. Neutralization efficacy was reported as the reciprocal of the highest serum dilution that resulted in at least 50% inhibition of outgrowth of EBV-infected lymphocytes. *Measurement of in vivo infection:* NA	Immunization of rabbits with the non-splicing gp350 variant resulted in high titers of anti-gp350 IgGs, with animals receiving the dose in Freund’s adjuvant developing a stronger response that those immunized with the dose in Alum. Both groups developed high titers of EBV nAbs.	This study was the first to produce a recombinant non-splicing gp350 variant free of the gp220 isoform. The generated protein was immunogenic, and led to the second EBV vaccine clinical trial reported, published in 2007 by Moutschen et al. (see **Table 2**).
18. Cox, 1998 (60) Immunization of common marmosets with Epstein-Barr virus (EBV) envelope glycoprotein gp340: effect on viral shedding following EBV challenge *(gp350)*	(a) NHP challenge model, common marmosets, adults (*Callithrix jacchus*); *sex not reported* (n = 5)	*Platform:* Monomeric protein *Antigen and dose*: 30µg of recombinant gp340 produced in C-127 cells *Adjuvant*: Alum (1:3 ratio of alum to protein)	*Control(s)*: Alum only (n = 6) *Immunization schedule*: Three intramuscular injections at week (-12, -8 -4). *Sample collection schedule*: Week (-12, 0, 2, 4, 8, 12, 14, 16, 20, 24, 28, 32, 37, 41, 45, 50) *Virus challenge*: Two oral injections of 3x10^4^ ID_50_ M81 EBV at week (0, 12). *Study duration*: 50 weeks	*Antibody assay(s)*: ELISA (gp340), and IFA for VCA/virus lytic antigens (VLA) on EBV-infected P3HR1 cells, using sera from immunized marmosets. *In vitro neutralization assay(s)*: None reported. *Measurement of in vivo infection:* EBV-specific PCR amplification from whole mouth fluid.	In the first experiment, immunization of adult marmosets with gp340 reduced viral load/shedding in the buccal cavity upon challenge when compared to injection with alum alone, but the immunization did not result in sterile immunity. Similar results were observed in the neonate experiment. However, neonates with seropositive parents, both in immunized and unimmunized groups, displayed indicators of EBV infection prior to challenge, suggesting natural infection occurred before EBV challenge.	As previous marmoset challenge studies, the overall results of this study suggest gp340 alone is not sufficient to induce sterile immunity, although it might be useful in reducing viral loads and potentially EBV disease. The results of the neonate experiment also provide further support for the common marmoset challenge model as an EBV model more closely resembling human EBV infection, where natural infection can occur and be transmitted from parents to children.
	(b) NHP challenge model, common marmosets, neonates *(Callithrix jacchus); sex not reported* (n = 3 from seronegative parents; n = 5 from seropositive parents)	*Platform*: Monomeric protein *Antigen and dose*: 5µg of recombinant gp340 produced in C-127 cells *Adjuvant*: Alum (1:3 ratio of alum to protein)	*Control(s)*: Alum (n = 3 from seronegative parents, n = 3 from seropositive parents). *Immunization schedule*: Three intramuscular injections at week (-12, -8 -4). *Sample collection schedule*: Week (0, 4, 9, 12, 16, 20, 24) *Virus challenge*: One oral injection of 1.5x10^4^ ID_50_ M81 EBV at week (0). *Study duration*: 24 weeks	*Antibody assay(s)*: Indirect IFA for VCA/VLA on EBV-infected P3HR1 cells, using sera from immunized marmosets, but results not shown. *In vitro neutralization assay(s)*: None reported. *Measurement of in vivo infection:* As above.		
19. Mackett, 1996 (61) *Immunization of common marmosets with vaccinia virus expressing Epstein-Barr virus (EBV) gp340 and challenge with EBV* *(gp350)*	NHP challenge model, common marmoset *(Callithrix jacchus); sex not reported* (n =4)	*Platform*: Vaccinia viral vector (Western Reserve [WR] strain) *Antigen and dose*: One dose of 5 x 10^7^ PFU and a second dose of 2 x 10^8^ PFU of recombinant vaccinia virus expressing gp340 (vMA1) *Adjuvant*: None	*Control(s)*: “empty” vaccinia virus (vTK-16; n = 3), PBS (n = 4). *Immunization schedule*: Two intradermal injections at week (-10, -5) *Sample collection*: Various timepoints after immunization and before challenge, after challenge, and after immunosuppression, over a period of 2.5 years. *Virus challenge*: 2 oral injections of 5 x10^4^ ID_50_ M81 EBV at week (0, 12). Between weeks 36-40 and 83-87, animals were immunosuppressed with a 32-day course of Cyclosporin A. *Study duration*: 2.5 years	*Antibody assay(s)*: ELISA (gp340), and direct IFA for VCA and early antigens on EBV-infected P3HR1 cells, using sera from immunized marmosets. *In vitro neutralization assay(s)*: None reported. *Measurement of in vivo infection*: Slot-blot (results not shown) and EBV-specific PCR amplification from whole mouth fluid.	Although the vaccine elicited anti-gp340 IgGs in immunized marmosets prior to EBV challenge, all animals eventually developed anti-gp340 responses following challenge that did not differ across treatment groups. Similarly, VCA responses were observed in immunized marmosets prior to challenge, but after challenge all animals developed similar VCA responses; after first immunosuppression VCA responses increased in all groups, but were unaffected after the second round. Persistent early antigen responses were observed in 1/4 of PBS and 2/3 vTK-16-treated animals, and sporadically in 2/4 PBS and 1/3 vTK-16-treated animals; while only 1/4 vaccine-treated animals developed sporadic early antigen responses, even after immunosuppression. Finally, PCR analyses detected DNA in 42% of samples collected from vaccinated animals, and 67% of samples collected from control animals, after immunosuppression.	The study used antibody responses against early antigen as a surrogate marker for virus replication, and DNA positivity in mouth fluids as a marker for virus burden. The vaccine was able to achieve a reduction of both markers in immunized marmosets when compared to control treatments, even after immunosuppression. This suggests that a gp340 vaccine might be able to reduce viral loads and potentially EBV disease in immunized humans, but that on its own is not sufficient to provide sterile immunity. However, it is important to note that the vaccinia strain used (WR strain), has been shown not to be too immunogenic [see (62)]
20. Finerty, 1994 (63) *Immunization of cottontop tamarins and rabbits with a candidate vaccine against the Epstein-Barr virus based on the major viral envelope glycoprotein gp340 and alum* *(gp350)*	(a) NHP challenge model, cottontop tamarins (*Saguinus oedipus oedipus*); *sex not reported* (n =5)	*Platform*: Monomeric protein Antigen and dose: 50μg of recombinant gp340 produced in C-127 cells *Adjuvant*: Alum (1:3 ratio of alum to protein)	*Control(s)*: PBS with alum (n = 1). *Immunization schedule*: Four intramuscular injections at approximately day (-111, -81, -51, -21) *Sample collection*: Various timepoints before and after immunization, and after challenge *Virus challenge*: Two administrations of B95-8 EBV, one intraperitoneal and one intramuscular, at a dose known to induce lymphoma, at Day (0). *Study duration*: ~166 days	*Antibody assay(s)*: ELISA (gp340), and IFA for VCA (results not shown), using sera from immunized tamarins. *In vitro neutralization assay(s)*: Neutralization assay (transformation inhibition) in cord blood lymphocytes using unreported EBV strain, with sera from immunized tamarins. Neutralization efficacy was reported as log_10_ reduction in virus titer. *Measurement of in vivo infection:* Tumor emergence monitoring (external palpation and measurement of lymph nodes; bodyweight recording).	Immunization of cottontop tamarins with gp340 adjuvanted with alum elicited gp340-specific antibodies only after the 4^th^ dose, with only a single animal eliciting nAbs. Regardless, 3 out of 5 animals were protected against EBV-induced lymphoma upon EBV challenge.	The results of this study emphasize the importance of the use of different animal models when evaluating vaccine and adjuvant immunogenicity. Alum in tamarins did not result in a robust humoral response, unlike SAF-1 in previous studies cited by the authors, but in rabbits both alum and SAF-1 proved equally immunogenic. Despite the low humoral immunogenicity observed in tamarins, over 50% of them were protected from EBV-induced lymphoma, suggesting cellular immunity had a potential role in controlling cancer development.
	(b) Rabbits; *strain and sex not reported* (n =2)	*Platform*: Monomeric protein *Antigen and dose*: 50μg (high dose) or 5μg (low dose) recombinant gp340 produced in C-127 cells, each administered with one of two adjuvants. *Adjuvants*: Alum or SAF-1	*Control(s)*: None reported. *Immunization schedule*: Four intramuscular injections at approximately week (0, 2, 4, 6) *Sample collection*: At (Pre-bleed, Week 2, 4, 6, 8) *Virus challenge:* NA Study duration: ~56 days	*Antibody assay(s)*: ELISA (gp340), using sera from immunized rabbits. *In vitro neutralization assay(s)*: As above. *Measurement of in vivo infection:* NA	All immunized rabbits elicited anti-gp340 antibodies, at higher titers than marmosets, regardless of adjuvant. Similarly, all alum and SF-1-adjuvanted immunized rabbits generated nAbs.	
21. Ragot, 1993 (64) *Replication-defective recombinant adenovirus expressing the Epstein-Barr virus (EBV) envelope glycoprotein gp340/220 induces protective immunity against EBV-induced lymphomas in the cottontop tamarins* *(gp350)*	NHP challenge model, cottontop tamarins (*Saguinus oedipus oedipus*); *sex not reported* (n =4)	*Platform*: Adenovirus (serotype 5) *Antigen and dose*: 5x10^9^ PFU, 1x10^10^ PFU, and 2 x10^10^ PFU replication-defective recombinant adenovirus (serotype 5) expressing gp340/220 produced from HEK-293 cells *Adjuvant*: None	*Controls*: Non-recombinant adenovirus (n = 1), unimmunized (n = 1). *Immunization schedule*: Three intramuscular injections at Week (0, 5, 13) *Sample collection:* Pre-bleed and then weekly *Virus challenge*: two administrations of B95-8 EBV, one intraperitoneal and one intramuscular, at a 100% tumorigenic dose, at week (16). *Study duration*: ~26 weeks	*Antibody assay(s)*: ELISA (gp340/220), using plasma from immunized tamarins. *In vitro neutralization assay(s)*: Neutralization assay in cord blood lymphocytes, using unknown strain of EBV, with plasma from immunized tamarins. Neutralization efficacy was not observed and was therefore not reported. *Measurement of in vivo infection:* Tumor emergence monitoring (external palpation and measurement of lymph node tumors).	Tamarins immunized with gp340/220 adenovirus elicited gp340/220-specific antibodies, but these antibodies were unable to neutralize infection *in vitro*. Despite this, all immunized animals were protected against EBV challenge, while control animals developed lymphoma.	The results of this study suggest a recombinant adenovirus approach could be a promising gp340/220 vaccine platform to reduce or prevent EBV disease. However, the fact that the generated antibodies were not neutralizing suggests EBV-induced cancer development was mostly controlled *via* cellular immunity.
22. Madej, 1992 (65) *Purification and characterization of Epstein-Barr virus gp340/220 produced by a bovine papillomavirus virus expression vector system* *(gp350)*	(a) BALB/c mice; *sex not reported* (n = 5)	*Platform*: Monomeric protein *Antigen and dose*: 0.1ml of 200µg/ml of recombinant gp340/220 ectodomain produced in mouse C-127 cells. *Adjuvant*: Alum	*Controls:* None reported. *Immunization schedule*: Four subcutaneous injections at Day (0, 14, 28, 42) *Sample collection*: Not stated. *Virus challenge:* NA *Study duration*: Not stated, but likely ~56 days.	*Antibody assay(s)*: ELISA (gp340/220), using sera from immunized mice. *In vitro neutralization assay(s)*: Neutralization assay (transformation inhibition) in cord blood lymphocytes using unreported EBV strain, with sera from immunized mice. Neutralization efficacy was reported as endpoint titer. *Measurement of in vivo infection:* NA	Immunized mice adjuvanted with Freund’s adjuvant elicited higher levels of anti- gp340/220 antibodies than immunized mice adjuvanted with alum, and produced nAbs at titers similar to tamarins immunized with gp340 isolated from B-958 cells in previous studies that were protected against EBV-induced lymphoma.	The study shows that it is feasible to produce and purify recombinant gp340/220 ectodomain, which maintains its antigenicity, using bovine papillomavirus virus as an expression system in mammalian cells. The resulting product is immunogenic and could elicit nAbs in immunized mice when given with alum, and its efficacy *in vivo* was tested in tamarins in a companion publication, Finerty et al., 1992 (see below).
	(b) BALB/c mice; *sex not reported* (n = 5)	*Platform*: Monomeric protein *Antigen and dose*: 0.1ml of 40ug/ml of recombinant gp340/220 ectodomain produced in C-127 cells. *Adjuvant*: Complete Freund’s adjuvant for primary immunization, incomplete Freund’s adjuvant for boosters	*Controls:* None reported. *Immunization schedule*: Four intraperitoneal injections at day (0, 14, 28, 42) *Sample collection*: Not stated *Virus challenge:* NA *Study duration*: Not stated, but likely ~56 days	*Antibody assay(s)*: As above. *In vitro neutralization assay(s)*: As above. *Measurement of in vivo infection:* NA		
23. Finerty, 1992 (66) *Protective immunization against Epstein-Barr virus-induced disease in cottontop tamarins using the virus envelope glycoprotein gp340 produced from a bovine papillomavirus expression vector* *(gp350)*	*NHP*, Cottontop tamarins (*Saguinus oedipus oedipus*); *sex not reported* (n = 4)	*Platform*: Monomeric protein *Antigen and dose*: 50μg recombinant gp340 ectodomain produced in C-127 cells. *Adjuvant*: SAF-1	*Control*: Non-immunized (n = 2). *Immunization schedule*: Four intramuscular injections at Day (0, 10, 20, 30) *Sample collection*: At (prebleed, Day 0, 10, 20, 30, 40) *Virus challeng*e: EBV administration, strain and route unstated, at a 100% tumorigenic dose, at Day (40) *Study duration*: ~95 days	*Antibody assay(s)*: ELISA (gp340), using plasma from immunized tamarins. *In vitro neutralization assay(s)*: Neutralization assay (transformation inhibition) in cord blood lymphocytes using unreported EBV strain, with plasma from immunized tamarins. Neutralization efficacy was reported as log_10_ reduction in virus titer. *Measurement of in vivo infection:* Tumor emergence monitoring (external palpation and measurement of enlarged lymph nodes and tumors).	Most immunized tamarins generated nAbs, that had efficacy paralleling the titers of anti-gp340 antibodies (higher anti-gp340 antibody producers had higher neutralizing activity). In turn, animals that generated nAbs were protected against EBV-induced lymphoma.	The study tests the *in vivo* vaccine efficacy of a recombinant gp340 ectodomain protein extensively characterized in a companion publication, Madej et al., 1992 (see above), for the first time. Administered with SAF-1 adjuvant, the protein induced nAbs and proved protective against EBV-induced lymphoma.
24. Zhang 1991 (67) *Mapping of the epitopes of Epstein-Barr virus gp350 using monoclonal antibodies and recombinant proteins expressed in Escherichia coli defines three antigenic determinants.* *(gp350)*	Rabbits; *strain and sex not reported* (n = not stated)	*Platform*: Monomeric protein *Antigen and dose*: 250µg beta-galactosidase fusion gp350 proteins covering various gp350 regions (nucleotides 1782 to 2307, 3576 to 4266, 3069 to 4146, or 2301 to 4287, of the BamHI L fragment of B95-8 EBV) produced in *E. coli*. *Adjuvant*: Complete Freund’s adjuvant for primary immunization, incomplete Freund’s adjuvant for booster	*Control*: Beta-galactosidase protein (n = not stated) *Immunization schedule*: Two immunizations at Day (0, 30). First immunization was administered both intradermally and subscapularly; second immunization was administered intradermally. *Sample collection*: At (Prebleed, Week 2, 4, 6) *Virus challenge:* NA *Study duration*: ~6 weeks	*Antibody assay(s)*: IFA (B95-8 cells), and dot blot immunoassay (gp350 protein, B95-8 cell lysates), using sera from immunized rabbits. *In vitro neutralization assay(s)*: Neutralization assay (transformation inhibition) in cord blood lymphocytes using B95-5 virus, and neutralization assay in Raji cells using P3HR1 virus, with sera from immunized rabbits. Infection was measured by transformation and EBNA IFA in cord blood lymphocytes, and early antigen IFA in Raji cells. Neutralization efficacy was reported as the presence or absence of transformation, and the presence or absence of positive EBNA1 IFA staining. *Measurement of in vivo infection: NA*	Although anti-gp350 antibodies were generated by immunization with the various truncated gp350 proteins, no nAbs were elicited.	The study produced and characterized various forms of truncated gp350 protein in bacteria with the purpose of mapping gp350 epitopes. When they tested the immunogenicity of some of these proteins, they found that they elicited no nAb activity, which was not unexpected as none had been recognized in dot blot assays by a known nAb, F29-167-A10. The authors suspect this to be the result of lack of post-translational modifications in the bacterial system, namely glycosylation. While we now know that glycosylation is indeed important for the functionality of gp350 nAb epitopes, none of their tested truncations included the now known major neutralizing epitope.
25. Morgan, 1989 (68) *Validation of a first-generation Epstein-Barr virus vaccine preparation suitable for human use* *(gp350)*	NHP, Cottontop tamarins (*Saguinus oedipus oedipus*); *sex not reported* (n =4)	*Platform*: Monomeric protein *Antigen and dose*: 50μg gp340 isolated from the membrane fraction of B95-8 cells *Adjuvant:* SAF-1 adjuvant containing 250μg threonyl muramyl dipeptide and 2.5%v/v copolymer	*Control*: Non-immunized (n = 1). *Immunization schedule*: 5 subcutaneous injections at day (0, 14, 28, 42, 56) *Sample collection*: Day (0, 14, 28, 42, 56, and 70) *Virus challenge*: EBV administration, strain and route unstated, 100% carcinogenic dose (10^5.3^ tissue culture transforming units), at day (70). *Study duration*:120 days	*Antibody assay(s):* ELISA (gp340) using sera from immunized tamarins. *In vitro neutralization assay(s)*: Neutralization assay (transformation inhibition) in human cord blood lymphocytes using unreported EBV strain with sera from immunized tamarins. Neutralization efficacy was reported as the presence or absence of transformation, and whether 1ml of sample neutralized >10^5^ lymphocyte transforming units of virus. *Measurement of in vivo infection:* Measurement of emergent tumors; measurement of EBV levels in the blood by *in vitro* transformation assay (co-culturing PBMCs from infected tamarins with human cord blood lymphocytes).	The vaccine was immunogenic, eliciting high titers of anti-gp340 antibodies, and protected 50% of the animals against virus infection and 100% against EBV-induced lymphoma.	This study used a new purification process to isolate gp340 from B95-8 cell membrane (automated FPLC). While the resulting vaccine was very successful in eliciting nAbs and preventing EBV-induced tumorigenesis in immunized tamarins, the overall results indicate that this formulation is not sufficient to prevent infection *in vivo*.
26. Emini, 1989 (69) *Vero cell-expressed Epstein-Barr virus (EBV) gp350/220 protects marmosets from EBV challenge* *(gp350)*	NHP, Common marmosets (*Callithrix jacchus*); *sex not reported* (n =4)	*Platform*: Monomeric protein *Antigen and dose*: 100µg recombinant gp350/220 produced in Vero cells delivered in one of two adjuvants *Adjuvant*: Alum or Freund’s adjuvant (complete for primary immunization, incomplete for boosters)	*Controls*: Alum (n = 2), or Freund’s adjuvant (n = 2). *Immunization schedule*: Thre intramuscular injections at Month (0, 1, 2). *Sample collection*: at Months (0, 1, 2, 3, 4, 5, 6, 7, 8, 9) *Virus challenge*: One injection of 8 log_10_ transforming units of B95-8 EBV, route unstated, at Month (3). *Study duration*: 9 months	*Antibody assay(s):* ELISA (gp350/220) using sera from immunized marmosets. *In vitro neutralization assay(s)*: Neutralization assay in Loukes cells, using B95-8 EBV, with sera from immunized marmosets (measured by IFA). Neutralization efficacy was reported as the highest serum dilution that yielded a positive neutralization. *Measurement of in vivo infection:* Measurement of antibody titers (IFA) against early antigen and VCA	All the marmosets immunized with gp350/220 in Freund’s adjuvant elicited high titers of anti-gp350/220 antibodies, compared to very low titers in marmosets immunized with gp350/220 in alum. Only one of the marmosets immunized with gp350/220 in alum had nAbs as tested *in vitro*, while two from the gp350/220 in alum group had nAbs. However, when it came to *in vivo* protection, protection was only conferred to 50% of marmosets immunized with gp350/220 in alum, compared to none in the gp350/220 in Freund’s adjuvant group. Thus, the presence of both total anti-gp350/220 antibodies or nAbs did not correlate with *in vivo* protection.	This study tested gp350/220 produced in mammalian cells as a vaccine in two different adjuvants, alum and Freund’s adjuvant. Neither formulation was able to provide sterile immunity to challenged immunized marmosets, although alum-adjuvanted marmosets performed better than Freund’s adjuvant despite displaying a very limited anti-gp350/220 antibody response. Regardless, no correlation was observed between total anti-gp350/220 antibodies or nAbs and *in vivo* protection, thus results are inconclusive.
27. Morgan, 1988 (70) *Recombinant vaccinia virus expressing Epstein-Barr virus glycoprotein gp340 protects cottontop tamarins against EB virus induced malignant lymphomas* *(gp350)*	NHP, Cottontop tamarins (*Saguinus oedipus oedipus*); *sex not reported* (n =4)	*Platform*: Vaccinia virus (Wyeth strain or WR strain) *Antigen and dose*: 20µl of 3.9x10^9^ PFU/ml (by scarification) or 2x10^9^ PFU/ml (intradermally) recombinant vaccinia virus expressing gp340, Wyeth strain, or 20µl of 5x10^9^ pfu/ml (by scarification) recombinant vaccinia virus expressing gp340, laboratory (WR) strain *Adjuvant:* None	*Controls*: Non-immunized (n = 4). *Immunization schedule*: For Wyeth strain immunizations, either one intradermal injection at Week (0; n = 2) or two applications by scarification at Week (0, 2; n = 2); for WR strain immunizations, either one application by scarification at Week (0; n = 2) or two applications by scarification at Week (0, 14; n = 2). *Sample collection*: Every two weeks after primary immunization *Virus challenge*: EBV administration, strain and route unstated, at 10^5.3^ transforming units of EBV (100% tumorigenic dose), six weeks after immunization (unclear whether after primary or secondary immunization). *Study duration*: ~110 days	*Antibody assay(s):* ELISA (gp340) and radioimmunoassay (gp340) using sera from immunized tamarins. *In vitro neutralization assay(s)*: Performed with sera from immunized tamarins, but conditions not reported. Neutralization efficacy was not explicitly reported. *Measurement of in vivo infection:* Tumor emergence monitoring (physical palpation and estimation of tumor size).	None to low gp340-specific antibodies were detected by ELISA and radioimmunoassay in all immunized tamarins. Importantly, no nAbs were detected in tamarins immunized with the Wyeth strain, and only very low nAb levels were detected in tamarins immunized with the WR strain (not shown). When it came to *in vivo* protection, neither group was fully protected from EBV-driven cancer, but more tamarins were protected in the WR strain group (3/4) than in the Wyeth strain (1/4).	This study explored the use of two different types of vaccinia viruses as gp340 vaccine platforms. The overall anti-gp340 antibody response and nAb responses were low to nonexistent in immunized tamarins, but partial protection was achieved. While there was no correlation between this protection and the observed levels of anti-gp340 antibodies and nAbs, a potential positive correlation was observed with the levels of anti-vaccinia antibodies (not discussed here). Thus, the mechanism behind the observed protection is unclear, although the authors speculate that it might be cell-mediated.
28. Morgan, 1988 (71) *Prevention of Epstein-Barr (EB) virus-induced lymphoma in cottontop tamarins by vaccination with the EB virus envelope glycoprotein gp340 incorporated into immune-stimulating complexes* *(gp350)*	NHP, Cottontop tamarins (*Saguinus oedipus oedipus*); *sex not reported* (n =4)	*Platform*: Nanoparticle *Antigen and dose*: <2 to 5µg of ISCOMS complexed to gp340 produced in B95-8 cells *Adjuvant:* ISCOM	*Controls*: Non-immunized (n = 4). *Immunization schedule*: Two subcutaneous injections of 2-5µg gp350-complexed ISCOMs at Week (0, 2), and 1 subcutaneous injection of <2µg gp340-complexed ISCOMs at Week (4) *Virus challenge*: EBV administration, strain and route unstated, at 10^5.3^ transforming units of EB, at Week (6). *Sample collection*: Unstated. *Study duration*: ~102 days	*Antibody assay(s):* ELISA (gp340) using sera from immunized tamarins. *In vitro neutralization assay(s)*: Neutralization assay (transformation inhibition)in cord blood lymphocytes using unreported EBV strain, with sera from immunized tamarins. Neutralization efficacy was not explicitly reported. *Measurement of in vivo infection:* Tumor emergence monitoring (external palpation and measurement of lymph node tumors).	Immunization of tamarins with gp340-complexed ISCOMs resulted in the generation of anti-gp340 antibodies as well as *in vitro* nAb activity (not shown). Importantly, all immunized tamarins were fully protected against EBV challenge.	This was the first study to use ISCOMs as an EBV glycoprotein-delivery system. Results were very encouraging and support the use of ISCOMs as an EBV vaccine platform.
29. Emini, 1988 (72) *Antigenic analysis of the Epstein-Barr virus major membrane antigen (gp350/220) expressed in yeast and mammalian cells: implications for the development of a subunit vaccine.* *(gp350)*	New Zealand white rabbits; *sex not reported* (n = 6)	*Platform*: Monomeric protein Antigen: 700µg of gp350/220 produced in yeast cells, or 200µg of gp350/220 produced in mammalian cells (either GH3 or Vero cells) *Adjuvant*: Freund’s adjuvant	*Control:* 20µg (n = 2) or 100µg (n = 4) of gp350/220 obtained from B95-8 cells. *Immunization schedule*: Three intramuscular injections at Month (0, 1, 2) *Sample collection*: Two weeks following last injection *Virus challenge*: NA *Study duration*: ~3.5 months	*Antibody assay(s):* ELISA (gp350/220) using sera from immunized rabbits. *In vitro neutralization assay(s)*: Neutralization assay in Loukes cells, using B95-8 EBV, with sera from immunized rabbits (measured by IFA), with and without complement. Neutralization efficacy was reported as the reciprocal of the highest serum dilutions at which complete neutralization was observed. *Measurement of in vivo infection: NA*	While all immunized rabbits produced anti-gp350/220 antibodies, only rabbits immunized with gp350/220 produced in mammalian cells generated detectable nAbs. The addition of complement in neutralization assays had varying effects on neutralization depending on the source of gp350/220.	This study was the first to directly compare the properties and immunogenicity of gp350/220 protein produced in yeast versus mammalian cells. Results from the characterization experiments (not discussed here), and most importantly from the immunogenicity experiments, suggest that glycoprotein post-translational modifications originating from the producer cell type play a critical role in antigenic presentation. Results from this study also suggest yeast is not a suitable cell type for EBV glycoprotein expression for vaccine purposes.
30. David, 1988 (73) *Efficient purification of Epstein-Barr virus membrane antigen gp340 by fast protein liquid chromatography.* *(gp350)*	BALB/c mice; *sex not reported* (n = 4)	*Platform:* Monomeric protein *Antigen and dose*: 10µg of gp340 isolated from the membrane fraction of B95-8 cells *Adjuvan*t: SAF-1	*Control*: None reported. *Immunization schedule*: Five subcutaneous injections at Day (0, 14, 28, 42, and 56) *Sample collection*: Day (0, 14, 28, 42, 56, 70) *Virus challenge*: NA *Study duration*: 70 days	*Antibody assay(s):* ELISA (gp340) using sera from immunized mice. *In vitro neutralization assay(s)*: Neutralization assay (transformation inhibition) in cord blood lymphocytes using unreported EBV strain, with sera from immunized mice. Neutralization efficacy was not explicitly reported. *Measurement of in vivo infection: NA*	Immunization of mice with this vaccine resulted in the production of high titers of anti-gp340 antibodies. High sera neutralizing activity was also reported, but results were not shown.	In this study the authors sought to establish a new method (FPLC-based) for purifying gp340 from B95-8 cells for vaccine purposes, due to the fact that gp340 purified using immunoaffinity chromatography using nAb 72A1 did not confer sterilizing protection (Epstein et al., 1986). The results of the study show that gp340 purified in this way and used as a vaccine with SAF-1 adjuvant is immunogenic, but the fact that no results were shown for neutralization assays makes the true immunogenic properties of this vaccine difficult to assess as is.
31. Epstein, 1986 (74) *Not all potently neutralizing, vaccine-induced antibodies to Epstein-Barr virus ensure protection of susceptible experimental animals* *(gp350)*	*NHP*, Cottontop tamarins (*Saguinus oedipus oedipus*); *sex not reported* (n = 4)	*Platform*: Nanoparticle *Antigen and dose*: 275µg of gp340 isolated from B95-8 cells, delivered in liposomes *Adjuvant*: 5x10^10^ *Bordatella pertussis* cells (first immunization only)	*Control*: Non-immunized (n = 2). *Immunization schedule*: Six intraperitoneal injections at Day (0, 14, 28, 42, 56, and 70) *Sample collection*: Prebleed and one bleed following each immunization, exact schedule unclear. *Virus challenge*: A single dose of 10^5.3^ transforming units of B95-8 EBV, delivered in an intramuscular and an intraperitoneal injection, at Day (105). *Study duration*: ~175 days	*Antibody assay(s):* ELISA (gp340) and IFA, using sera from immunized tamarins. *In vitro neutralization assay(s)*: Neutralization assay (transformation inhibition) in cord blood lymphocytes using unreported EBV strain, with sera from immunized tamarins. Neutralization efficacy was reported on whether 1ml serum neutralized >100,000 lymphocyte transforming units of virus. *Measurement of in vivo infection:* Tumor emergence monitoring (external palpation and measurement of lymph node tumors).	Despite all immunized tamarins developing high titers of anti-gp340 antibodies and high sera neutralizing activity, no immunized animals were protected from the EBV challenge.	This study utilized immunoaffinity chromatography to isolate gp340 from B95-8 cells to be delivered in a liposome-based EBV vaccine. While a previous liposome formulation incorporating gp340 isolated *via* a molecular weight-based method fully protected tamarins from EBV challenge, no tamarins in this study were protected against challenge despite displaying high levels of sera neutralizing activity. Several potential reasons were given to explain the failed results, including (i) gp340 must undergo denaturation during SDS-PAGE preparations before immunization; (ii) essential gp340 molecules are not isolated by the 72A1 nAb; and (iii) elution of gp340 from the 72A1 column at pH 11.5 may destroy important antigenic carbohydrate structure. This study highlights the importance of testing the efficacy of EBV vaccine candidates *in vivo*, and the potential dangers of relying on *in vitro* efficacy data alone.
32. Mackett, 1985 (75) *Recombinant vaccinia virus induces neutralising antibodies in rabbits against Epstein-Barr virus membrane antigen gp340* *(gp350)*	Rabbits; *strain and sex not reported* (n = 2)	*Platform*: Vaccinia virus (WR strain) *Antigen and dose*: 10^8^ PFU of vaccinia virus expressing gp340 *Adjuvant*: None	*Control*: Empty vaccinia (n = 1). *Immunization schedule*: Two intradermal injections at Week (0, 4) *Sample collection*: Prebleed and at Week (4,8) *Virus challenge*: NA *Study duration*: 3 months	*Antibody assay(s):* IFA (B95-5 and W91 cells) using sera from immunized rabbits. *In vitro neutralization assay(s)*: Performed with sera from immunized rabbits, but conditions not reported. Neutralization efficacy was reported as the relative strength of neutralization. *Measurement of in vivo infection:* NA	Immunization of rabbits with gp340-expressing vaccinia elicited high titers of EBV-specific antibodies as well as nAbs.	This was the first study to use a viral vector as an EBV vaccine platform. Although the immunization experiments were done in very few animals, the obtained results supported the use of a vaccinia-based EBV vaccine.
33. Epstein, 1985 (76) *Protection of cottontop tamarins against Epstein-Barr virus-induced malignant lymphoma by a prototype subunit vaccine* *(multiple antigens, gp350)*	*(a) NHP*, Cottontop tamarins (*Saguinus oedipus oedipus*); *sex not reported* (n = 2)	*Platform*: Infected cell membrane *Antigen and dose*: Purified B95-8 cell membranes containing 2,500 units of gp340. *Adjuvant*: None	*Control(s)*: Non-immunized (n = 2). *Immunization schedule*: Eight intraperitoneal injections at Week (0, 2, 4, 6, 8, 10, 12, 14) *Sample collection schedule*: NA *Virus challenge*: A single dose of 10^5.3^ lymphocyte transforming units of B95-8 EBV (timing NA, route unclear). *Study duration*: NA	*Antibody assay(s)*: IFA (B95-8 cells) and ELISA (gp340), using sera from immunized tamarins. *In vitro neutralization assay(s)*: Neutralization assay (transformation inhibition) in cord blood lymphocytes using unreported EBV strain, with sera from immunized tamarins. Neutralization efficacy was reported on whether 1ml serum neutralized >10,000, 100,000 or >100,000 lymphocyte transforming units of virus. *Measurement of in vivo infection*: Tumor emergence monitoring while alive (external palpation and measurement of lymph node tumors); necropsy analysis of tumor lesions in spleen, liver, kidney, gut wall, adrenals, and lymph nodes.	Purified EBV-infected cell membrane, which likely contained multiple glycoproteins and not only gp340, elicited high titers of nAbs that completely protected NHP from lymphoma after 8 immunizations. gp340 purified by size and delivered *via* liposomes did elicit nAbs, but challenged animals that generated nAbs still developed transient lesions.	The results of this study were regarded as evidence that gp340 might serve as an important EBV vaccine target. Indeed, immunization with gp340 delivered *via* liposomes resulted in *in vitro* nAb activity. However, not all immunized animals were fully protected against disease, and 2/3 that were fully protected were immunized with infected cell membrane that likely contained additional glycoproteins. In addition, the immunization regimen (8-17 immunizations) required to achieve the observed level of nAb activity is not a feasible or practical realistic vaccine regimen. Regardless, the small number of animals used in the study is not statistically powered to allow an accurate assessment.
	*(b) NHP*, Cottontop tamarins (*Saguinus oedipus oedipus*); *sex not reported* (n = 4)	*Platform*: Nanoparticle *Antigen and dose*: gp340 isolated from B95-8 cell membranes, delivered in liposomes (dose NA) *Adjuvant*: None	*Control(s)*: None reported. *Immunization schedule*: NA *Sample collection schedule*: NA *Virus challenge*: A single dose of 10^5.3^ lymphocyte transforming units of B95-8 EBV (timing NA, route unclear). *Study duration*: NA			
	*(c) NHP*, Cottontop tamarins (*Saguinus oedipus oedipus*); *sex not reported* (n = 2)	*Platform*: Nanoparticle *Antigen and dose*: 2,250 units of gp340 isolated from B95-8 cell membranes, delivered in liposomes *Adjuvant*: None	*Control(s)*: Non-immunized (n = 2) *Immunization schedule*: Seventeen intraperitoneal injections at Week (0, 2, 4, 6, 8, 10, 12, 14, 16, 18, 20, 22, 24, 26, 28, 30, 32). *Sample collection schedule*: NA *Virus challenge*: A single dose of 10^5.3^ lymphocyte transforming units of B95-8 EBV (timing NA, route unclear). *Study duration*: NA			
34. Morgan, 1984 (77) *Comparative immunogenicity studies on Epstein-Barr virus membrane antigen (MA) gp340 with novel adjuvants in mice, rabbits, and cottontop tamarins* *(gp350)*	(a) Female BALB/c mice (n = 4)	*Platform*: Nanoparticle *Antigen and dose*: 70 or 15 units of gp340 isolated from B95-8 cells, delivered as (a) liposomes; (b) liposomes incorporating the lipid A fraction from *Escherichia coli* lipopolysaccharide; or (c) liposomes mixed with *Bordatella pertussis* cells. *Adjuvant*: lipid A, or 2x10^9^ killed *Bordatella pertussis* cells.	*Control(s):* 70 or 15 units of purified gp340 isolated from B95-8 cells in complete Freund’s adjuvant (n = 4). *Immunization schedule:* Variable according to treatment. Liposomes alone were delivered intravenously; liposomes with lipid A were delivered either intraperitoneally (at two different doses) or intravenously; liposomes with *Bordatella pertussis* cells were delivered intraperitoneally; control was delivered intraperitoneally. All groups were boosted twice at monthly intervals, except for liposomes with *Bordatella pertussis* which were only boosted once. *Sample collection schedule:* Two weeks after each injection *Virus challenge:* NA *Study duration:* ~100 days	*Antibody assay(s)*: Binding assay to radiolabeled gp340; immunoprecipitation/SDS-PAGE analysis of B95-8 and M-ABA cell lysates (results for P3HR-1 and QIMR-WIL cells not shown). *In vitro neutralization assay(s)*: NA *Measurement of in vivo infection*: NA	In mice, liposome formulations with lipid A were superior to any other formulations in eliciting anti-gp340 antibody titers, and the only treatment to elicit a response with the low gp340 dose. All liposome formulations elicited higher responses than purified gp340 in Freund’s adjuvant. Immune sera successfully immunoprecipitated gp340 from B95-8 and M-ABA cells. Although the data was not shown, reported responses in rabbits to liposomes alone were nonexistent, and only observed in animals that received either liposomes with lipid A, or purified gp340 in Freund’s adjuvant after boosts. Similar to mice, liposomes with lipid A was the only treatment to elicit a response with the low gp340 dose. In tamarins, immunizations with liposomes incorporating lipid A also performed better than liposomes alone, and the generated antibodies were found to be neutralizing. Immune sera immunoprecipitated gp340 from B95-8 cells.	This study explored the role of different factors that could influence immunogenicity of antigens presented in liposomes, including animal species, adjuvants, and route of vaccine delivery. The study found that gp340 delivered as liposomes incorporating lipid A as an adjuvant performed better than any other gp340 treatment independent of the immunization route, a pattern that was conserved across the species tested. This led the authors to establish the gp340 liposome/lipid A formulation as a potential candidate to move forward in future EBV vaccine studies. This was also the first study to test an EBV vaccine candidate in cottontop tamarins, which is used as an EBV challenge model in subsequent studies. While the study emphasizes the importance of testing vaccine candidates in various animal models, unclear details in immunization dose and schedules, and differences in immunization schedules across species, complicate comprehensive analysis.
	(b) Sandy-Lop rabbits; *sex not reported* (n = unspecified)	*Platform*: Nanoparticle *Antigen and dose*: 300-500 units of gp340 isolated from B95-8 cells, delivered as (a) liposomes; or (b) liposomes incorporating lipid A. *Adjuvant*: lipid A.	*Control(s):* 300-500 units of purified gp340 isolated from B95-8 cells in complete Freund’s adjuvant. (n = unspecified). *Immunization schedule:* Variable according to treatment. Liposomes alone and liposomes with lipid A were delivered intravenously; control was delivered subcutaneously. All groups were boosted at 3-4 weekly intervals. *Sample collection schedule:* Two weeks after each injection. *Virus challenge:* NA *Study duration:* NA	*Antibody assay(s)*: Unspecified. *In vitro neutralization assay(s)*: NA *Measurement of in vivo infection*: NA		
	*(c) NHP*, male and female, Cottontop tamarins (*Saguinus oedipus oedipus*) (n = 4)	*Platform*: Nanoparticle *Antigen and dose*: 1000-2000 antigen units of gp340 isolated from B95-8 cells, delivered as (a) liposomes mixed with *Bordatella pertussis* cells; or (b) liposomes incorporating lipid A. *Adjuvant*: 10^10^ killed *Bordatella pertussis* cells, or lipid A	*Control(s):* None reported. *Immunization schedule:* Both treatments were delivered intraperitoneally, and groups were boosted every 3-9 weeks. *Sample collection schedule:* 2-5 weeks after each boost. *Virus challenge:* NA *Study duration:* NA	*Antibody assay(s)*: Binding assay to radiolabeled gp340; immunoprecipitation/SDS-PAGE analysis of B95-8 cell lysates. *In vitro neutralization assay(s)*: Neutralization assay (transformation inhibition) in cord blood lymphocytes using unreported EBV strain, with sera from immunized tamarins. Neutralization efficacy was reported as the dilution that neutralized 1000 transforming doses of EBV. *Measurement of in vivo infection*: NA		
35. North, 1982 (78) *Purified Epstein-Barr virus Mr340,000 glycoprotein induces potent virus-neutralizing antibodies when incorporated in liposomes* *(gp350)*	Female BALB/c mice (n = 4)	*Platform:* Monomeric protein and nanoparticle *Antigen and dose*: 70-80 units of gp340 isolated from B95-8 cells delivered as (a) purified protein mixed with Complete Freund’s Adjuvant; (b) purified protein mixed with *Bordatella pertussis* organsisms; or (c) liposomes mixed with *Bordatella pertussis* organsisms. Adjuvant: Freund’s adjuvant, or 2x10^9^ *Bordatella pertussis* organisms	*Controls*: None reported. *Immunization schedule*: Two intraperitoneal injections at Week (0, 6) *Sample collection:* At Week (4, 10) *Virus challenge*: NA *Study duration*: 10 weeks	*Antibody assay(s)*: Radioimmunoassay (gp340); immunoprecipitation analysis of B95-8 and QIMR-WIL cell lysates. *In vitro neutralization assay(s)*: Neutralization assay (transformation inhibition) in cord blood lymphocytes using M-ABA strain EBV, with sera from immunized mice. Neutralization efficacy was reported as the 50% endpoint dilution. *Measurement of in vivo infection*: NA	Immunization of mice with gp340 elicited high anti-gp340 antibody titers when the antigen was delivered as liposomes with *Bordatella pertussis*, but no response was observed when delivered as purified protein. Reactive sera specifically immunoprecipitated gp340 from B95-8 and QIMR-WIL cell lysates. Furthermore, pooled sera from mice immunized with the liposomal formulation showed robust neutralizing activity against M-ABA strain EBV.	This study provided the first evidence of preparation of purified gp340 from EBV-infected cell membranes as a subunit vaccine, delivered in a liposomal formulation (nanoparticle). These encouraging results opened the way for various future studies focused on gp340 liposomal formulations as EBV vaccine candidates.
36. Thorley-Lawson, 1979 (79) *A virus-free immunogen effective against Epstein-Barr virus* *(multiple antigens)*	Rabbit; *sex not reported* (n = 1)	*Platform*: Monomeric protein (multiple) *Antigen and dose*: 100µg of membrane proteins from P3HR-1 cells *Adjuvant*: Complete Freund’s adjuvant	*Controls*: 100µg of membrane proteins from Ramos cells (n = 1) *Immunization schedule*: Three subcutaneous and intravenous injections over the course of two months. *Virus challenge*: NA Sample collection: 10 days after last injection *Study duration*: 8-10 weeks	*Antibody assay(s)*: NA *In vitro neutralization assay(s)*: Neutralization assay (transformation inhibition) in B lymphocytes from adult donors using B95-8 EBV, with sera from immunized rabbits. Neutralization efficacy was reported as the number of wells transformed at different sample dilutions. *Measurement of in vivo infection*: NA	Immunization with P3HR-1 membrane proteins elicited high titers of nAbs that neutralized infection in adult B lymphocytes, in contrast with immunization from Ramos cell membrane proteins, which did not elicit neutralizing sera.	The study reported the first successful large-scale production of DNA-free immunogens from the plasma membrane of EBV producer cell lines. This protein mix elicited high titers of nAbs, further cementing the fact that membrane antigens are important EBV vaccine targets, and identifying multiple antigens. However, the number of animals used is too small to infer meaningful conclusion.

**Table 4:** Characteristics of the clinical studies included in the systematic review.

First author, year of publication, and title *(glycoprotein[s] targeted)*	Location, Phase and Vaccine	Study endpoints and Study population features	Study Groups and Features	Measurement of vaccine response	Trial Outcomes	Overall assessment
1. Rees, 2009 (80) *A Phase I trial of Epstein-Barr virus gp350 vaccine* *for children with chronic kidney disease awaiting* *transplantation* *(gp350)*	United Kingdom, Phase I, open fixed-dose escalation Recombinant gp350 protein (splice-site mutated to prevent formation of 220 isoform) produced in CHO cells	Safety and immunogenicity 16 EBV-negative children with chronic kidney disease (CKD) awaiting renal donation (median age 8.9 years, range 1.4-17.6 years)	*Groups*: Immunization with 12.5µg gp350 adjuvanted with alhydrogel (n=6); 25µg gp350 adjuvanted with alhydrogel (n=10). *Immunization Schedule*: Three three- or four-weekly subcutaneous injections. A fourth vaccination was offered at weeks 30 to 32 for EBV-negative children who were not transplanted and had low anti-gp350 antibody levels. *Sample collection Schedule*: Variable (not defined) *Study Duration*: ~40 weeks	*Antibody assays*: ELISA (gp350). *In vitro neutralization*: Competitive ELISA (gp350) against anti-gp350 neutralizing antibody (not reported) using sera from immunized individuals. *Measurement of in vivo infection*: ELISA (EBNA1, VCA), immunoblot (antigens not reported), PCR (EBNA1, BamHI-W).	The vaccine was well-tolerated at both dose levels, with only two systemic reactions occurring at the 25µg dose level. Two patients received only two vaccinations (group(s) unclear). In the 25µg dose level group, 3/10 patients received a fourth vaccination at week 30-32. All evaluable patients in the 12.5µg dose level (n=4) and 25µg dose level (n=9) displayed a detectable anti-gp350 response. Only 1/4 patients in the 12.5µg dose level 3/9 patients in the 25µg dose level displayed nAb responses. The n=1 evaluable patient that received a fourth vaccination experienced a rapid and substantial increase in anti-gp350 antibodies, and the emergence of nAb, which were absent before. Four patients (group(s) unclear) acquired EBV infection during the study period, and seven others were reported to display high levels of EBV genomic loads 26 weeks post-transplant (off-trial), with levels similar to unvaccinated transplant recipients that had been EBV-negative prior to transplant, which were higher that unvaccinated transplant recipients that had been EBV-positive prior to transplant. One episode of EBV-related PTLD was reported in a patient in the 12.5µg dose level soon after transplantation, 50 weeks after the first vaccination.	Results from this study confirm this gp350-based vaccine at the current formulation and doses is safe and tolerable in children with CKD awaiting transplantation. The vaccine was immunogenic in all evaluable patients, but only 4/13 generated nAbs. EBV infection was detected in various patients both before and after transplantation, with one case of EBV-associated PTLD. Although the study was not designed to test vaccine efficacy, these results suggest the vaccine does not influence post-transplant EBV loads or prevent PLTD at its current state. Thus, new formulations and/or vaccination schedules might be needed to successfully lower EBV loads post-transplant the occurrence and severity of EBV-associated PTLD in this patient population.
2. Sokal, 2007 (81) *Recombinant gp350 vaccine for infectious* *mononucleosis: a Phase 2, randomized, double-* *blind, placebo-controlled trial to evaluate the* *safety, immunogenicity, and efficacy of an Epstein-* *Barr virus vaccine in healthy young adults* *(gp350)*	Belgium, Phase II, double-blinded placebo-controlled randomized Recombinant gp350 protein (splice-site mutated to prevent formation of 220 isoform) produced in CHO cells	Efficacy at preventing infectious mononucleosis (primary); efficacy at preventing primary EBV infection, and immunogenicity (secondary) 181 EBV-negative 16–25-year-old individuals (51.1% male and 97.8% Caucasian, 20.6 years mean age, in vaccine group; 53.8% male and 96.7% Caucasian, 20.5 years mean age, in placebo group)	*Groups*: Immunization with 50µg gp350 adjuvanted with ASO4 (n=90); 0.5mg Alum (placebo, n=91). *Immunization Schedule*: Three intramuscular injections at month (0, 1, 5) *Sample collection Schedule*: Month (0, 1, 5, 6, 19) *Study Duration*: 18-21 months	*Antibody assays*: ELISA (gp350). *In vitro neutralization*: Competitive ELISA (gp350) against anti-gp350 neutralizing antibody 72A1 using sera from immunized individuals. *Measurement of in vivo infection*: IFA (VCA IgG and IgM) and ELISA (VCA IgG and IgM); health monitoring for infectious mononucleosis symptoms.	The vaccine was well-tolerated, and no severe adverse events associated with the vaccine were reported. In the placebo group, 18/90 of participants became infected, nine who developed infectious mononucleosis and nine who displayed asymptomatic infection. In the vaccine group, 13/90 participates became infected, two who developed infectious mononucleosis (both before the third immunization) and eleven who displayed asymptomatic infection. Infectious mononucleosis rates were found to be significantly different between the two groups, and vaccine efficacy at preventing infectious mononucleosis was reported to be 78% (intention to treat population). 1 month after the third dose, anti-gp350 antibodies were detected in 98.7% of participants who hadn’t already become EBV-positive; it was only at this point that anti-gp350 antibody levels in vaccinees exceeded those seen after natural infection. Neutralizing antibodies as measured by 72A1 competitive ELISA peaked at this time as well, detected in 69.86% of participants.	Results from this study suggest a gp350-based vaccine can be effective at preventing infectious mononucleosis in healthy young adults (78% efficacy). While the study was not designed to measure long-term efficacy, the results suggest the vaccine might protect against infectious mononucleosis even after eighteen months post-primary immunization. In this system, three doses might provide full protection against disease; however, the vaccine was not successful at preventing primary EBV infection.
3. Moutschen, 2007 (82) *Phase I/II studies to evaluate safety and immunogenicity of a* *recombinant gp350 Epstein–Barr virus vaccine in healthy adults* *(gp350)*	Belgium, Phase I, double-blind randomized Recombinant gp350 protein (splice-site mutated to prevent formation of 220 isoform) produced in CHO cells	(a) Safety (primary) and immunogenicity (secondary) 36 EBV-negative and 31 EBV-positive 18–24-year-old young adults (59.7% male, 94.0% Caucasian)	*Groups*: Immunization with 50µg gp350 adjuvanted with ASO4, EBV-negative (EBV-/AS04, n=20); 50µg gp350 adjuvanted with ASO4, EBV-positive (EBV+/AS04, n=15); 50µg gp350 adjuvanted with Alum, EBV-negative (EBV-/Alum, n=15); 50µg gp350 adjuvanted with Alum, EBV-positive (EBV+/Alum, n=16). *Immunization Schedule*: Three intramuscular injections at month (0, 1, 6) *Sample collection Schedule*: Month (0, 1, 2, 6, 7) *Study Duration*: 7 months	*Antibody assays*: ELISA (gp350). *In vitro neutralization*: Neutralization assay (transformation inhibition) in B lymphocytes from adult donors, with sera from immunized individuals. *Measurement of in vivo infection*: IFA and ELISA (assay details not provided).	Both vaccine formulations were found to be safe and well-tolerated, with only one serious adverse event reported in EBV+/AS04 that was deemed potentially related to vaccination. Anti-gp350 antibodies were detected in all vaccinees one month after third immunization. Neutralizing antibodies were detected in 100% of EBV-/AS04, 71.4% in EBV+/AS04-, 44.4% in EBV-/Alum, and 55.5% of EBV+/Alum vaccinated subjects one month after third immunization.	Results from both studies confirm that recombinant soluble gp350 as a non-adjuvanted or adjuvanted (Allum or AS04) vaccine is safe and immunogenic in both EBV-negative and EBV-positive healthy adults. The vaccine stimulated both humoral and cellular immunity (not discussed here). However, despite all participants generating anti-gp350 antibodies after the third immunization, not all vaccinees developed neutralizing antibodies. Four subjects in the Phase I trial, and six subjects in the Phase I/II trial became EBV positive as detected during a 7-month follow-up.
	Belgium, Phase I/II, double-blind randomized Recombinant gp350 protein (splice-site mutated to prevent formation of 220 isoform) produced in CHO cells	(b) Safety (primary) and immunogenicity (secondary) 81 EBV-negative 18–36-year-old adults (55.6% male and 98.8% Caucasian)	*Groups*: Immunization with 50µg gp350 adjuvanted with Alum (n=27); 50µg gp350 adjuvanted with AS04 (n=27); 50µg non-adjuvanted gp350 (n=27). *Immunization Schedule*: Three intramuscular injections at month (0, 1, 6) *Sample collection Schedule*: Month (0, 1, 2, 6, 7) *Study Duration*: 7 months	*Antibody assays*: ELISA (gp350). *In vitro neutralization*: Neutralization assay (transformation inhibition) in B lymphocytes from adult donors, with sera from immunized individuals; competitive ELISA (gp350) against anti-gp350 neutralizing antibody 72A1 using sera from immunized individuals. *Measurement of in vivo infection*: IFA (VCA IgG and IgM) and ELISA (VCA IgG and IgM).	All vaccine formulations were found to be safe and well-tolerated, with only 1 serious adverse event reported in the Alum-adjuvanted group, but it was not suspected to be related to vaccination. Anti-gp350 antibodies were detected in all vaccinated subjects 1 month after third immunization, but non-adjuvanted vaccine resulted in lower titers. Neutralizing antibodies were detected in 60.9% of Alum-adjuvanted vaccinees, 50% of AS04-aduvanted vaccinees, and 32% of non-adjuvanted vaccinees, as measured by neutralization assay, and in 81.8% of Alum-adjuvanted vaccinees, 71.4% of AS04-aduvanted vaccinees, and 50% of non-adjuvanted vaccinees, as measured by competitive ELISA.	
4. Gu, 1995 (83) *First EBV vaccine trial in humans using recombinant vaccinia virus expressing the major membrane antigen* *(gp350)*	China, Phase I Live recombinant vaccinia virus (*Tien Tan strain*) expressing gp220-340 produced in 2BS human cells	(a) Safety and immunogenicity 11 EBV-positive and vaccinia-exposed adults	*Groups*: Immunization with 10^8^ pfu/ml of vaccinia virus expressing gp220-340 (n=11). *Immunization Schedule*: Once, by scarification at two sites (single arm) *Sample collection Schedule*: Month (0, 1) *Study Duration*: 1 month	*Antibody assays*: IFA and ELISA (vaccinia, EBV-gp350, and EBV VCA). *In vitro neutralization assays*: NA *Measurement of in vivo infection:* IFA and ELISA antibody assays.	3/11 vaccinees had fever reactions and local lesions, with seroconversion to vaccinia. 8/11 had weak responses with local redness, swelling and itching, but no seroconversion to vaccinia. There were no detectable differences in anti-gp350 antibody before and after immunization in all vaccinees.	Results from the adult study suggest that previous exposure to vaccinia virus impaired immune response against the vaccine. Vaccinia non-exposed juveniles and infants displayed an overall strong response to the vaccine. However, based on the infant study, the vaccine might be effective at reducing rate of EBV infection, but not at fully preventing infection.
		(b) Safety and immunogenicity 6 EBV-positive and vaccinia non-exposed 8–9-year-old juveniles	*Groups*: Immunization with 10^7^ pfu/ml of vaccinia virus expressing gp220-340 (n=6). *Immunization Schedule*: Once, by a single scarification *Sample collection Schedule*: At month (0, 1) Study Duration: 1 month	*Antibody assays*: IFA and ELISA (vaccinia, EBV-gp350, and EBV VCA). *In vitro neutralization assays*: Neutralization assay using patient sera in Raji cells. *Measurement of in vivo infection:* IFA and ELISA antibody assays.	Vaccinees developed local lesions with no fever. 5/6 developed antibodies to both vaccinia and gp350 one-month post-immunization. EBV nAbs were detected in sera of all vaccinees between 1:20 and 1:80 dilutions.	
		(c) Safety, immunogenicity, and efficacy in preventing primary EBV infection 19 EBV-negative and vaccinia non-exposed 1-3-year-old infants	*Groups*: Immunization with 10^7^ pfu/ml of expressing gp220-340 (n=9); unvaccinated (n=10). *Immunization Schedule*: Once, by a single scarification *Sample collection Schedule*: At month (0, 1, 6, 16) *Study Duration:* 16 months		Vaccinees developed local lesions with no fever. All vaccinees developed anti-vaccinia antibodies. 8/9 vaccinees developed anti-gp350 antibodies one month after vaccination and nAbs in sera detected between 1:40 and 1:60 dilutions. 3/9 vaccinated infants and 10/10 unvaccinated infants became infected with EBV by Month 16 post-immunization.	

### 2.3 Quality Assessment

The quality of all selected pre-clinical studies was assessed using a checklist of 18 items (**Table 5**) that were modified and expanded from (84), which was developed based on the Collaborative Approach to Meta-Analysis of Animal Data from Experimental Stroke (CAMARADES).

**Table 5:** Quality assessment checklist.

Criteria	Additional description for clarity when needed
1. Clear objectives and methodology of proposed study	
2. Masked assessment of outcome (analysis blinded)	
3. Details of formulation, dosage, and route of vaccine being administered	
4. Negative immunization controls included	
5. Positive immunization controls included	
6. Timing of specimen collection stated	*Did the study describe the schedule of blood and/or organ collection?*
7. Duration of the study stated	*Did the study explicitly state the period of observation or indicate the sacrifice of the study animals?*
8. Toxicity evaluation of the vaccination	*Did the study evaluate the safety of the vaccine tested in the study animals?*
9. Sample size calculation or N=5/group	
10. Retrievable neutralization assay data	
11. Titer/infectivity information for virus used for neutralization provided	*Did the study provide any infectivity information of the EBV batch used for in vitro neutralization assays or for in vivo challenge experiments?*
12. Neutralization concentrations/dilutions provided	*Did the study provide information regarding serum/plasma/antibody concentration or dilution used in neutralization assays?*
13. Neutralization timepoint provided	*Did the study indicate the timepoint of antibody/serum/plasma sample(s) used in reference to the study schedule?*
14. Statistical analysis performed	
15. Statistical analysis methods provided	
16. Statement of compliance with regulatory requirements	
17. Statement regarding possible conflict of interest	
18. Publication in a peer-reviewed journal	

These 18 items were divided into primary (#1-15) and secondary (#16-18) quality criteria. Each primary quality criterion carried a score of one point. In addition, a study was awarded two points for 3/3 of the secondary criteria met, one point for 2/3, and no points for 0-1/3, giving a possible total of 17 points. Because four of the primary criteria (#11-13, #15) did not apply to all studies, studies with total scores of 13 or above were considered very high quality. Studies with scores 10-12 were high quality, 7-9 were moderate quality, 5-8 were poor quality, and 0-4 were very poor quality. Tabulated results for each study analyzed are presented in **Table S3**, and the overall quality distribution of the studies was graphed and presented in **Figure 4D**.

### 2.4 Ethical Statement

No institutional review board approval was required for this study.

## 3 Results

### 3.1 Study Selection

Our electronic database search yielded a total of 5,614 unique articles. Of these, 36 pre-clinical studies and 4 clinical studies met the criteria for inclusion in the systematic review (**Figure 2**). The pre-clinical studies were conducted between 1979 and 2020 (**Table 3**) and the clinical studies were conducted between 1995 and 2009 (**Table 4**). Most studies were conducted in the United States and the United Kingdom (**Figure 3**).

### 3.2 Description of Included Pre-Clinical Studies

In the 36 pre-clinical studies included in our systematic review, gp350 was the most commonly used individual immunogen, tested in a total of 33 studies (**Figure 4A**); gH/gL and gB were individually tested in 3 studies each. When multivalent combinations were tested, undefined viral membrane antigens were used in 3 studies, gp350-gH/gL and gp350-gB combinations were used in 2 studies each, and additional antigen combinations were used in 1 study each (**Figure 4A**). Vaccines were most commonly delivered as monomeric proteins (12 studies); other delivery platforms included nanoparticles (8 studies), virus-like particles (VLPs; 5 studies), multimeric proteins and vaccinia virus (4 studies each), and infected cell membranes (2 studies), as well as peptides, DNA, measles virus, and adenovirus (1 study each) (**Figure 4B**). Mice were the most commonly used animal for immunogenicity assessments (15 studies), followed by rabbits and cotton-top tamarins (10 studies each), common marmosets (3 studies), and cynomolgus macaques (2 studies), as well as rats and rhesus macaques (1 study each) (**Figure 4C**). Of these studies, 8 utilized cotton-top tamarins and 3 utilized common marmosets as EBV challenge models (**Figure 4C**).

Most pre-clinical studies (13) were rated as moderate quality, 11 were rated as poor quality, 5 were rated as high quality, 4 were rated as very high quality, and 3 were rated as very poor quality (**Table S3** and **Figure 4D**).

### 3.3 Description of Included Clinical Studies

All 4 clinical studies included in our systematic review used gp350 as the clinical immunogen (**Table 4**). The first study (83), a Phase I study, used vaccinia virus (*Tien Tan strain*) to deliver the immunogen (**Table 4**). The second (82), a Phase I/II study, and third (81), a Phase II study, used a monomeric gp350 protein formulation with Adjuvant System 04 (AS04) (**Table 4**). The most recent study (80), a Phase I study, used a monomeric gp350 protein formulation of the same origin as the others but adjuvanted with alum alone (**Table 4**). None of these clinical studies led to the prevention of EBV infection/sterile immunity.

## 4 Discussion

Despite four decades of vaccine research on EBV since its discovery in 1964 (7), there is still no clinically approved prophylactic vaccine against EBV infection. In this systematic review, we sought to examine every pre-clinical and clinical EBV prophylactic vaccine study focused on humoral immunity performed up to June 20, 2020 (36 pre-clinical trials and 4 clinical trials), to provide insight on the four main factors preventing the successful development of an effective EBV prophylactic vaccine (1): undefined correlates of immune protection (2); lack of an appropriate animal model to test vaccine efficacy; (3) lack of knowledge regarding the ideal EBV antigens for vaccination; (4) and lack of knowledge regarding the ideal vaccine delivery platform to present relevant EBV antigens.

### 4.1 Correlates of Immune Protection Against Primary EBV Infection

The correlates of immune protection against primary EBV infection are not well-defined, partly due to a lack of an appropriate animal model to study EBV infection and test vaccine efficacy, as discussed below in Section 4.2. Thus, pre-clinical vaccine efficacy has been measured primarily using *in vitro* neutralization assays as a surrogate for *in vivo* protection. However, in our assessment, we found that there are no clear standards for performing neutralization assays, defining vaccine efficacy, or reporting outcomes.

Before the turn of the century, *in vitro* neutralization was assessed *via* transformation assays, in which primary human lymphoid B cells were exposed to EBV previously incubated with immune serum or plasma and then allowed to transform and immortalize over several weeks (**Table 3**) (85). A reduction in or lack of transformation indicated the presence of nAbs in the serum/plasma sample. The advent of fluorescent recombinant EBV (86–88) allowed the emergence of a simpler and faster method that enables the neutralization assay to be performed on various virus-susceptible cells, including established epithelial and B cell lines—eliminating the required use of primary B cells—with a maximum turnaround of three days after infection. Thus, most pre-clinical studies, beginning with Ruiss et al. in 2011 (56) (**Table 3**), have utilized a flow cytometry-based assay that was optimized by Sashihara et al. in 2009 to measure infection by quantifying the number of fluorescent cells in a given sample (89). Although these assays can provide invaluable insights, they are no substitute for *in vivo* studies and cannot provide true correlates of immune protection. Indeed, in the clinical trials analyzed in our study (**Table 4**), the presence of nAbs could still be identified *via in vitro* assays in vaccinees who later became EBV-positive. Moreover, we found that there is extensive variability in the type of cell lines used in neutralization assays (e.g., Raji, HEK-293, SVKCR2, Akata, AGS) and in how outcomes are reported (e.g., % neutralization, % infected cells, IC_50_ titer) (**Table 3**). We also found that most studies did not provide any information regarding the infectivity of the EBV batch used for the assays (e.g., infectivity curve or virus titer) or the target level of infection used as the negative control for neutralization; based on our assessment, only 14/36 studies appropriately reported virus titer (**Table S3**). Similarly, we found that only 21/36 studies appropriately reported the immune serum/plasma/antibody concentration or dilution used for neutralization assays (**Table S3**).

Competitive ELISA against a known nAb has also been used as a surrogate *in vitro* neutralization assay (**Table 3**). Although this assay provides important information regarding the types of known neutralizing epitopes a vaccine can target, it serves more as a complementary assay as it does not consider all potential targeted epitopes or any neutralizing mechanisms that can otherwise be probed using the traditional virus-based neutralization assay. Therefore, our review demonstrates a need to establish clear *in vitro* EBV neutralization protocols, reporting standards, and guidelines so that universal surrogate correlates of immune protection can be compared across *in vitro* efficacy studies. Moving forward, we propose that all studies perform virus-based neutralization assays using a full panel of established EBV-susceptible cell lines, when possible, especially if the vaccine tested targets multiple antigens that might be important for the infection of different cell types. In addition, viral titer information for the EBV batch used and the serum/plasma/antibody concentration in which the virus is incubated should be clearly reported; otherwise, an accurate assessment of vaccine efficacy and immune protection correlates is not possible.

Most studies in our analysis focused on assessing serum IgG responses, but it is important to note that mucosal IgA responses might also be a relevant source of protective nAbs, given that EBV is transmitted *via* the oral mucosa. In our assessment, we found that only 1/36 of the pre-clinical studies, and none of the clinical studies, assessed vaccine-induced IgA responses. In the one study that did assess IgA responses, these responses were measured in the serum [(45), **Table 3**]. A recent study assessing the presence of EBV glycoprotein-specific antibodies in patients with EBV-associated nasopharyngeal carcinoma, found that nAb activity in both epithelial and B cells was most strongly correlated with glycoprotein-specific IgGs rather than IgAs in the serum. However, IgA-related nAb activity might be most relevant in the saliva, as saliva is the first line of defense against EBV once the virus enters the oral mucosa. Since IgGs can also be found in saliva, both salivary IgAs and IgGs might be important to provide protection at the site of EBV infection (90). A recent study using the EBV-homologue rhesus lymphocryptovirus (rhLCV) model (described in Section 4.2 below) showed that oral transfer of glycoprotein-specific monoclonal IgGs can provide partial protection against rhLCV infection in rhesus macaques, demonstrating the importance of antibodies in the oral mucosa in infection prevention (91). While vaccine-induced salivary Ig responses might be difficult to study in small animal models, future pre-clinical non-human primate (NHP) studies and clinical studies should also assess both salivary and serum IgA/IgG responses. This will lead to better understanding of correlates of immune protection.

### 4.2 Animal Model(s) to Test Vaccine Efficacy and Explore Correlates of Immune Protection

EBV is a human-tropic virus, making it difficult to study *in vivo*. Rodents, rabbits, and various NHPs have been historically used to test EBV vaccine immunogenicity (**Table 4** and **Figure 4C**). However, only the cotton-top tamarin and common marmoset have been used to test *in vivo* vaccine efficacy. The cotton-top tamarin was the first animal to be established as an experimental model for EBV infection and the development of EBV-driven lymphoma (92). In 1985, the model was used for the first time to test the *in vivo* efficacy of a gp350 vaccine candidate (76). After that, 7 more studies used the model to test the efficacy of other gp350 vaccine candidates (**Figure 4C**), with the last study in 1994, as the model was abandoned in the early 2000s due to the endangered status of the species. Perhaps the greatest utility of this model was that it offered a direct readout of virus-associated disease (i.e., lymphoma) that was easy to measure in a relatively short timeline. However, the translatability of this model is limited because it did not truly recapitulate EBV infection in humans in terms of infection route, disease pathogenesis, or immune response to infection. The common marmoset, established as an experimental model of EBV infection in parallel with the cotton-top tamarin, was first used to test *in vivo* vaccine efficacy in 1989 (69, 93). Compared to the cotton-top tamarin model, this model is more translatable to human EBV infection (94, 95); nevertheless, only 2 more studies used it to test vaccine efficacy in the late 1990s, and it has since been abandoned for unclear reasons.

As a consequence of this dearth of *in vivo* models, *in vitro* neutralization assays using sera antibodies from immunized animals have been used as the primary vaccine “efficacy” readout in most pre-clinical EBV vaccine studies (**Table 3**), as described above. Humanized mice with a reconstituted human immune system are also susceptible to EBV infection of B cells, and like cotton-top tamarins develop lymphoma upon infection, providing a relatively quick readout of disease. However, this model suffers from the same drawbacks as cotton-top tamarins in terms of dissimilarities to human EBV infection; their epithelial cells are not susceptible to infection and their use is more suited for testing therapeutics rather than prophylactics. Rabbits have also been reported as susceptible to EBV infection (96–99), but no EBV-associated pathologies have been shown, and as humanized mice, their epithelial cells are not susceptible to infection (100). Similarly, Chinese tree shrews were recently reported to be susceptible to EBV B cell infection (101), but a previous study by the same group suggests that their epithelial cells are not infected by EBV (102). Both rabbits and tree shrews have yet to be used as challenge models to test EBV vaccine efficacy.

A promising alternative to test the efficacy of EBV vaccine candidates is to use rhesus macaques infected with rhesus lymphocryptovirus (rhLCV) as a surrogate for EBV infection. rhLCV shares high amino-acid homology with EBV (103), and rhLCV genes can complement the viral activities of EBV orthologs (104). Importantly, rhLCV infection in rhesus macaques recapitulates most aspects of EBV infection in humans, including the routes of infection, use of homologous glycoproteins to infect the host, disease establishment, and host immune responses (105). Furthermore, rhLCV-infected rhesus macaques that become immunosuppressed develop virus-associated malignancies of both epithelial and lymphoid origin, similar to immunosuppressed EBV-infected humans (106). The similarities between EBV and rhLCV were underscored in a recent study from the McGuire group, in which AMMO1, an EBV-specific nAb, was able to reduce rhLCV infection in rhesus macaques that had been passively infused with the nAb and subsequently challenged with rhLCV (107). Thus, the rhLCV infection model in rhesus macaques offers an opportunity to test the efficacy of EBV vaccines in the form of surrogate rhLCV vaccines in both immunocompetent and immunosuppressed settings, and future EBV vaccines studies should consider using this model to validate their *in vitro* findings.

The field could also re-evaluate the use of the common marmoset as an EBV challenge model. Early EBV studies using this model demonstrated the establishment of EBV infection in challenged animals and uncovered pathogenic similarities between common marmoset and human EBV infection (93). For example, infected common marmosets sporadically shed the virus in the oral mucosa and can transmit it to EBV-naïve animals through close contact, similar to how EBV transmission is thought to occur in humans (60). Infected common marmosets also share similar EBV antibody kinetics to infected humans (94). With the support of more modern EBV diagnostic methods and the rapid growth of common marmosets as a research NHP, this model has the potential to provide a powerful tool for testing EBV vaccine efficacy.

Use of NHPs such as rhesus macaques and common marmosets in EBV research will also facilitate the study of salivary antibodies to arrive at a better understanding of correlates of immune protection. However, one commonly overlooked caveat to the use of NHPs in this field, whether to test general vaccine immunogenicity or vaccine efficacy in a challenge model, is that all NHPs should be screened for infection with EBV-homologue LCVs. Cynomolgus macaques, rhesus macaques, and common marmosets are all naturally infected by species-specific LCVs (108), and as is the case in humans, infection is usually pervasive due to the high transmissibility of these viruses. This presents a problem due to the potential antibody cross-reactivity between the viruses, which could skew the assessment of any anti-EBV response induced in immunized animals. In our evaluation, only 1/16 studies that used NHPs acknowledged this issue [(50), **Table 3**], and no NHP study reported the use of LCV-negative animals. Thus, it is critical that moving forward all NHPs should be screened for native LCVs prior to any EBV vaccine immunogenicity studies. Development of specific-pathogen-free NHP colonies worldwide that are LCV-negative would be ideal.

### 4.3 Vaccine Antigen(s) Required to Elicit a Sufficiently Protective Response Against Primary EBV Infection

To date, there is still no consensus as to which glycoprotein(s) would serve as ideal vaccine antigens. Before the EBV glycoproteins were identified, early studies used whole virus or undefined membrane antigens from EBV-infected cells to begin exploring the general immunogenicity of EBV (109–111). By 1979, the analysis of purified cellular membranes from EBV-infected Raji cells allowed separation of various membrane-associated antigens with different molecular weights, which later became known as gp350/220, gB and gH (112). Despite this discovery and the identification of various nAbs specific to different glycoproteins in the 1980s and 2000s, most pre-clinical vaccine studies and all four clinical studies that followed focused on gp350 as the main vaccine target up until the 2010s, when other glycoproteins began to be seriously explored as additional potential vaccine antigens. The likely culprit for this focus seems to be the 1980 study by North, Morgan and Epstein, where neutralizing sera from rabbits immunized with whole virus was used to immunoprecipitate antigens from EBV producer cell lines, and gp350 was the only antigen precipitated at a detectable level (113). Furthermore, two gp350-specific nAbs were isolated and reported that same year (**Table 1**), strengthening the support for a gp350-based vaccine. Most gp350 vaccine studies that followed did result in the generation of gp350-specific antibodies with neutralizing capabilities *in vitro*, but no pre-clinical *in vivo* vaccine efficacy study has definitively shown protection against primary EBV infection. Ragot et al. (64) and Morgan et al. (70) demonstrated that gp350-expressing adenovirus and ISCOM-based gp350 nanoparticles fully protected cotton-top tamarins from developing EBV-induced lymphoma, respectively, but only n=4 animals were tested in each study (**Table 3**). Epstein et al. (76) similarly showed that cotton-top tamarins were fully protected from lymphoma when administered purified B95-8 membrane, but only n=2 animals were tested in this study. Thus, in these few cases showing positive results, too few experimental animals were tested to provide definitive conclusions, and Epstein et al. (76) also mentioned the presence of trace amounts of EBV in the administered vaccine product, which could have resulted in the emergence of nAbs against additional glycoproteins, skewing the results. Furthermore, there is no guarantee that protection from disease means primary infection did not take place; Morgan et al. (68) measured EBV infection in the blood and showed that although all cotton-top tamarins (n=4) were protected against lymphoma, only half were protected from infection (**Table 3**). Epstein et al. (76) demonstrated that cotton-top tamarins administered gp350 liposomes were ultimately protected against disease (n=2), but they developed transient lesions during the observation period (**Table 3**), suggesting that primary infection did take place.

This pattern continued in the clinical setting, and the first EBV vaccine clinical trial in 1995, which used a gp350 vaccinia-based vaccine, did not result in sterilizing immunity in vaccinated infants (83). Similar to the inconclusive results of the pre-clinical studies, this trial suggested that gp350 as a single vaccine antigen might not be sufficient to protect against EBV infection. Then, in 2000, the Delecluse group showed that recombinant EBV lacking gp350 could still infect both epithelial and B cells *in vitro* (114), suggesting that EBV could remain infectious even in the presence of gp350-specific nAbs. Nevertheless, three more clinical trials using gp350 vaccines were put forward in the 2000s (80–82). Although these vaccines reduced the incidence of IM in some cases, they did not provide sterilizing immunity against infection.

In the 2000s, several groups began to fully uncover the mechanisms of EBV entry in both epithelial and B cells, and the importance of the core fusion glycoproteins, gp42, gH/gL, and gB became apparent, as summarized in **Figure 1**. Our group and others have shown that gp350 binds complement receptor type 1 (CD35) (115) or type 2 (CD21) (116) on B cells and triggers endocytosis of the virions (30). Although this interaction is not essential as discussed above, it does enhance infection (114). Once the virus is bound to its target cell, the fusion machinery is required to achieve virus–cell fusion. gB is highly conserved among herpesviruses and is considered the core fusogen, achieving fusion through attachment to the host receptor neuropilin 1 (117). gB exists in two conformations, the pre- and the post-fusion states (118), and it is unclear which conformation might be best suited as a vaccine antigen (i.e., is more immunogenic) in the case of vaccines that rely on conformational epitopes; this remains an area of active investigation. In epithelial cells, the fusogenic activity of gB is triggered by the interaction of the gH/gL complex with the host ephrin A2 receptor (117, 119, 120). In B cells, gp42, in complex with gH/gL, activates gB after interacting with major histocompatibility complex (MHC) class II; in this way, the level of gp42 expression on the virion confers host cell specificity, promoting the infection of B cells and inhibiting the infection of epithelial cells (121, 122). Importantly, although it can mature and egress upon lytic induction, recombinant EBV lacking gH cannot infect epithelial cells, and recombinant EBV lacking gp42 or gH cannot infect B cells, suggesting that these glycoproteins are essential for EBV infection (37, 123). nAbs against these glycoproteins were also discovered between 1982 and 2000 (**Table 1**). Despite these discoveries, there have only been a few pre-clinical studies exploring non-gp350 glycoproteins as vaccine antigens (**Figure 4A**).

Given what we now know about the EBV infection process and how several glycoproteins collaborate to infect different types of target cells, we expect that the most effective vaccine strategy would be a multivalent approach targeting multiple important glycoproteins. Indeed, the fusion machinery constitutes the most obvious candidate to test in a multivalent EBV vaccine, but gp350 remains an important source of nAbs, and as such, it might still be an important vaccine target when used in combination with other antigens. Moreover, a recent study identified gp350 as the entry glycoprotein for T cells, which has implications for EBV-associated T and NK/T-cell lymphomas (124). Following this reasoning, the first glycoprotein combination vaccine was tested in 2008 by Lockey et al., which combined gp350 and gB (57). Although results were not conclusive on whether the combination was better than targeting gp350 alone, further combination studies that followed did provide support for a multivalent approach. For example, a recent pre-clinical study by the Cohen group reported better *in vitro* neutralization outcomes in mice after immunization with vaccines that combined the gH/gL and gp42/gH/gL complexes with gp350, compared to a vaccine that targeted gp350 alone; in the same study, a vaccine targeting the gp42/gH/gL complex resulted in better fusion inhibition outcomes than a vaccine targeting gH/gL complex alone (44). Similarly, in a recent study, our group reported that a multivalent vaccine incorporating gp350, gB, gp42, and gH/gL resulted in better neutralizing outcomes in immunized rabbits than a recombinant gp350 vaccine, with comparable results to immunization with UV-inactivated EBV (43). However, it is still not clear what combination of glycoproteins would best to prevent infection *in vivo* or whether incorporating all five glycoproteins is necessary. Thus, future studies should focus on identifying the required components of a multivalent EBV vaccine that can generate effective neutralizing activity both *in vitro* and *in vivo*. Indeed, ModernaTX Inc. has recently started recruiting participants for a Phase I clinical trial that will explore the safety and immunogenicity of their mRNA-based EBV vaccine, mRNA-1189, which targets multiple EBV glycoproteins (125–127).

While gp350, gB, gp42 and gH/gL have been the most studied entry glycoproteins, there are additional EBV envelope glycoproteins that could also prove to be important vaccine targets. BMRF2 has been reported to participate in viral entry by facilitating viral attachment to epithelial cells through its interactions with various host integrins (128, 129), and it has been implicated in cell-to-cell spread as well (130). There is data suggesting its potential to elicit nAbs (131), and it might be a relevant target if the virus escapes initial neutralization and breaches the oral mucosa, where it could spread from B cells to monocytes and to epithelial cells (132) *in vivo*. BDLF2 is co-expressed with BMRF2 (133) and thus might also be involved in cell-to-cell spread (134), but not much has been further uncovered about this glycoprotein. BDLF3/gp150 has been shown to bind to heparan sulfate proteoglyclans on the surface of epithelial cells, but this binding does not enhance infection (135), and its role in immune evasion may preclude any role in viral entry (136). BILF2 might be involved in glycoprotein transport (137), but otherwise is considered an orphan glycoprotein as no definite function has been uncovered for it (122). Future studies might reveal the potential utility of these additional glycoproteins as vaccine targets. Importantly, while the purpose of this review was to study previous prophylactic vaccine efforts focused on humoral immunity, we cannot ignore the possibility that a vaccine designed to target both humoral and cellular immunity could prove protective. Indeed, although we did not discuss the details, a few studies in our analysis [(46, 56, 57); **Table 3**] tested vaccines incorporating both glycoproteins and different targets of cellular immunity. The importance of these combinations remains to be tested *in vivo*. Furthermore, ideal vaccine regimen, dose, and route might differ between humoral and cellular immune targets, thus these parameters may be first tested separately for their immunogenicity before combining immune targets.

### 4.4 Ideal Vaccine Platform to Present Relevant EBV Antigen(s) in a Sufficiently Immunogenic Format

In our analysis, most pre-clinical trials (**Figure 4B**, **Table 3**) and clinical trials (**Table 4**) delivered EBV antigens as monomeric proteins, the most widely used platform in the earlier half of EBV vaccine research. Monomeric proteins are perhaps the least immunogenic of the vaccine platforms available, requiring the use of adjuvants to induce a robust immune response. The pre-clinical studies in our analysis used a wide variety of adjuvants, including aluminum hydroxide/alum, glucopyranosyl lipid A/GLA, stable emulsion/SE, SAF-1, and Freund’s adjuvant (**Table 3**) (138). In addition to low immunogenicity, another limitation to the use of monomeric proteins is that the higher-level protein structure of the antigens might be impaired in the monomeric state, inhibiting the induction of conformation-dependent nAbs (139). It is perhaps for these reasons that, after a lag in pre-clinical prophylactic EBV vaccine development between 1999 and 2008, several additional antigen delivery approaches were adopted to incorporate protein structural support and to provide the ability to present multiple antigens per unit. For example, two pre-clinical studies by the Snapper group explored the use of multimeric proteins [(48, 54), **Table 3**]. In both studies, the multimeric protein vaccines were more immunogenic than their corresponding monomeric protein vaccines. The use of self-assembling nanoparticles incorporating EBV glycoproteins has also been explored. The Cohen group (44, 50) showed that ferritin-based nanoparticles incorporating gp350, gH/gL, or gH/gL/gp42 were more immunogenic than the monomeric soluble forms of these glycoproteins, and the National Institute of Allergy and Infectious Diseases is currently recruiting participants to test the safety and immunogenicity of the gp350-ferritin-based nanoparticle in a Phase I clinical trial (140). Nanoparticles in the form of liposomes were also tested in the early stages of EBV vaccine research [(74, 76–78), **Table 3**], which focused on gp350. The platform was not pursued further, perhaps due to its ultimate inability to prevent lymphoma in cotton-top tamarins after viral challenge; however, as discussed in Section 4.3, this result may have occurred due to the use of gp350 as a single immunogen rather than insufficiency of the platform. Other recent studies have explored the use of VLPs, a special type of nanoparticle that resembles viruses in structure and, like other nanoparticles, can present multiple antigens or epitopes in a repetitive array, increasing antigen immunogenicity (141). Importantly, VLPs lack viral DNA, which is critical in the development of vaccines for oncogenic viruses such as EBV. The success of VLPs as a vaccine platform is underscored by the many licensed VLP-based vaccines on the market, including vaccines against human papillomavirus and hepatitis B and E. Five pre-clinical studies have investigated the use of VLPs for EBV, including VLPs produced from EBV-packaging cell lines (56), VLPs based on Newcastle disease virus (49), and VLPs produced from the hepatitis B virus core antigen (42). Although vaccines that present proteins in a multimeric form, whether as multimeric proteins or nanoparticles, are a better alternative to monomeric proteins, they might be expensive to produce in large quantities and still require adjuvants to achieve an adequate level of immunogenicity.

Viral vectors have the advantage of possessing intrinsic immune-stimulating capabilities and thus do not require administration with adjuvants; in addition, they can be combined with VLP platforms to produce VLPs *in vivo* (142–144). The 6 pre-clinical EBV vaccine studies using viral vectors tested measles virus (55), adenovirus (64), and vaccinia virus vectors (57, 61, 70, 75) (**Table 3**); the latter was also tested in one of the 4 EBV vaccine clinical trials [(83), **Table 4**]. In these studies, the vectors were engineered to express single antigens, but viral vectors with the necessary genetic insert capacity can be engineered to express multiple antigens. Measles virus and adenovirus vectors have good clinical track records, but they do not possess the genetic capacity to express multiple EBV antigens (145, 146). Vaccinia virus vectors, on the other hand, have the largest genetic insert capacity of all available viral vaccine vectors (>30 kb), and although the original vaccinia strains can pose serious health complications, the highly attenuated modified vaccinia Ankara (MVA) strain is safe even in immunocompromised populations and is highly immunogenic (147–149). Hence, future multivalent vaccine studies looking to use a viral vector-based platform should consider an MVA vaccine platform.

Although we did not encounter any studies within our search limits that utilized mRNA-based vaccines, the new Phase I clinical trial by ModernaTX Inc. that is currently recruiting participants to test a multivalent mRNA-based EBV vaccine will open the way for this technology to the field of EBV vaccine research (127). Having only recently been clinically tested at a large scale against SARS-CoV-2, questions about the response durability of mRNA-based vaccines remain (150), and whether this will be a successful approach for targeting multiple antigens in the context of EBV remains to be studied.

While not a concern for viral or nucleic-acid-based vaccines, an important consideration when producing protein antigens for vaccination is the choice of expression system. Post-translational modifications, such as glycosylation, are an important element that can affect antigen immunogenicity. Indeed, the Hayes group in 2015 studied the conformational requirements of gp350 to generate a nAb response [(51), **Table 3**], and found that glycosylation is essential to gp350 nAb generation, which can only properly be achieved in mammalian expression systems. This was previously demonstrated by two studies in 1991 and 1988 [(67, 72), **Table 3**] which explored expression of gp350 in bacteria and yeast, respectively, and found the resulting products incapable of generating nAbs, presumably due to the inability of these systems to perform complex glycosylation (151). Protein production in simpler systems such as bacteria and yeast do offer various advantages in terms of large-scale production feasibility, but unless glycosylation engineering approaches are applied in this context (152), mammalian cells remain the ideal production system for EBV glycoproteins.

A final consideration when it comes to vaccine platforms pertains to the feasibility of large-scale manufacturing, including processing, purification, and quality testing. An early EBV vaccine study by Epstein and his group [(74), **Table 3**] demonstrates the potential relevance of establishing appropriate processing methods. In this study, the authors used a protocol established by Randle et al., 1985 (153) to perform large-scale purification of gp350 from EBV producer cells using immunoaffinity chromatography; however, they found that the resulting product had a specific activity of 20% of that of a product prepared by molecular weight-based separation methods in small-scale, and failed to protect animals against EBV disease when delivered in liposomes. The authors attribute this to either the type of purification used resulting in antigen denaturation, or the isolated protein fraction not being immunogenic enough to confer protection as the previous formulation did. Today, biological manufacturing techniques have greatly improved, but the fact that different vaccine platforms possess different biochemical and structural qualities that require unique processing needs and quality testing should still be considered when designing a new vaccine that can be produced in enough quantity and with high enough quality for large-scale immunization (154). Thus, an ideal vaccine platform should not only be sufficiently immunogenic and protective, but also allow for the necessary infrastructure to facilitate future clinical translation.

### 4.5 Pre-Clinical Study Quality

Most of the pre-clinical studies included in our systematic review were of moderate, high, or very high quality (**Figure 4D** and **Table S3**); all pre-clinical studies of poor or very poor quality were performed before the year 2000. Although there was a clear shift toward higher-quality studies after 1999, our analysis demonstrated that there is still room for improvement. For example, none of the studies analyzed performed a blinded analysis, which would reduce potential bias and enhance scientific rigor. In addition, only 3 studies explicitly evaluated the toxicity of the vaccine tested (**Table S3**), which is essential for selecting vaccine candidates that are suitable for clinical use. As discussed in Section 4.1, it is particularly important to report neutralization assay details to accurately assess vaccine efficacy, including titer/infectivity information for the viruses used, but this was not the case for many of the analyzed studies. The use of both positive and negative control treatment groups and appropriately powered group sizes are also critical but were frequently not reported. Future studies should focus on these and other qualities (**Table S3**) to generate high-caliber studies with accurate, generalizable conclusions.

### 4.6 Limitations of the Study

While we aimed for our report to be as robust as possible, we do acknowledge a few limitations in our study. The small number of clinical trials included in our analysis, together with the variability in the quality (**Table S3**) and content of the pre-clinical trials, did not allow us to perform a meta-analysis or any additional meaningful statistical analysis or comparison between the studies, so our results lack statistical relevance. In addition, although our search was thorough to the best of our efforts, it is possible that we might have missed articles that fit our selection criteria.

Finally, while we structured our discussion towards developing a humoral-based vaccine to prevent primary EBV infection, this goal might be unrealistic. No sterilizing immunity has yet been achieved in pre-clinical or clinical studies against any herpesvirus. In the case of EBV, recent passive immunization studies in humanized mice (155) and in rhesus macaques using the rhLCV model (91, 107), did not achieve sterilizing immunity, although these studies were not focused on multivalent approaches. Cellular immunity might also be an important component to improve protective vaccine responses, which we did not consider in our review. Nevertheless, even if sterilizing immunity cannot be achieved against EBV *via* vaccination, an EBV vaccine might still be effective in reducing the rates of EBV diseases and their associated morbidities and mortality, such as is the case for the approved varicella zoster virus vaccines (156), and the EBV gp350-based vaccine tested in a Phase II clinical trial that was 78% successful in preventing infectious mononucleosis [(81), **Table 4**].

## 5 Conclusions

Our analysis of 36 pre-clinical studies and 4 clinical studies conducted over the last four decades strongly supports the use of a multivalent approach to develop an effective prophylactic EBV vaccine. In addition, testing *in vivo* vaccine efficacy in more robust animal models, such as the common marmoset and rhLCV-susceptible rhesus macaques, is expected to facilitate the establishment of standardized *in vivo* correlates of immune protection and the attainment of more generalizable and translatable data. Finally, our analysis suggests that new vaccine-developing studies should explore vaccine platforms that can enhance immunogenicity *via* multimeric approaches. We anticipate that evidence-based rational vaccine design, guided by the studies presented here, will yield an effective EBV vaccine that can finally be translated into the clinic to prevent more than 200,000 cases of cancer and numerous cases of IM and autoimmune disease each year.

## Data Availability Statement

The original contributions presented in the study are included in the article/**Supplementary Material**. Further inquiries can be directed to the corresponding author.

## Author Contributions

Conceived and designed the study: JO, GE, and LM. Performed the systematic review: JO, GE, LM, MM, and ER. Analyzed the data: JO, GE, LM, MM, and ER. Wrote the paper: JO, GE, LM, MM, and ER. All authors contributed to the article and approved the submitted version.

## Funding

This study was funded by the National Institute for Allergy and Infectious Diseases R56AI148295 grant to JO, and Department of Defense W81XWH-20-1-0401 Horizon Award to GE. The funding agencies were not involved in the design of the study, data analysis or interpretation, nor manuscript preparation or study publication.

## Conflict of Interest

The authors declare that the research was conducted in the absence of any commercial or financial relationships that could be construed as a potential conflict of interest.

## Publisher’s Note

All claims expressed in this article are solely those of the authors and do not necessarily represent those of their affiliated organizations, or those of the publisher, the editors and the reviewers. Any product that may be evaluated in this article, or claim that may be made by its manufacturer, is not guaranteed or endorsed by the publisher.
