# Supplementary material for: Four Decades of Prophylactic EBV Vaccine Research: A Systematic Review and Historical Perspective
Source: Front Immunol. 2022 Apr 14;13:867918. doi: 10.3389/fimmu.2022.867918 (PMC9047024; doi:10.3389/fimmu.2022.867918)
Supplement: Supplementary file 3 [file Table_3.docx]

**Table S3. Quality assessment of the pre-clinical studies included in the systematic review.**

|  | | Zhang et al., 2020 | Escalante et al., 2020 | Bu et al, 2019 | Zhao et al., 2018 | Perez et al., 2017 | Heeke et al., 2016 | Cui et al., 2016 | Ogembo et al., 2015 | Kanekiyo et al., 2015 | Servat et al., 2015 | Tanner et al., 2015 | Cui et al., 2013 | Mok et al., 2012 | Ruiss et al., 2011 | Lockey et al. 2008 | Wilson et al., 1999 | Jackman et al., 1999 | Cox et al. 1998 | Mackett et al., 1996 | Finerty et al., 1994 | Ragot et al., 1993 | Madej et al., 1992 | Finerty et al., 1992 | Zhang et al., 1991 | Morgan et al., 1989 |
| --- | --- | --- | --- | --- | --- | --- | --- | --- | --- | --- | --- | --- | --- | --- | --- | --- | --- | --- | --- | --- | --- | --- | --- | --- | --- | --- |
| Clear objectives and methodology of proposed study | | ✓ | ✓ | ✓ | ✓ | ✓ | ✓ | ✓ | ✓ | ✓ | ✓ | ✓ | ✓ | ✓ | ✓ | ✓ | ✓ | ✓ | ✓ | ✓ | ✓ | ✓ | ✓ | ✓ | ✓ | ✓ |
| Masked assessment of outcome (analysis blinded) | | NR | NR | NR | NR | NR | NR | NR | NR | NR | NR | NR | NR | NR | NR | NR | NR | NR | NR | NR | NR | NR | NR | NR | NR | NR |
| Details of formulation, dosage and route of vaccine being administered | | ✓ | ✓ | ✓ | ✓ | ✓ | ✓ | ✓ | ✓ | ✓ | ✓ | ✓ | ✓ | ✓ | ✓ | ✓ | ✓ | x | ✓ | ✓ | ✓ | ✓ | ✓ | ✓ | ✓ | ✓ |
| Negative immunization controls included | | ✓ | ✓ | NR | ✓* | ✓ | ✓ | ? | ✓ | ✓* | NR | NR | NR | ✓* | ✓ | ✓ | ✓* | NR | ✓ | ✓ | ✓* | ✓ | NR | ✓ | ✓ | ✓ |
| Positive immunization controls included | | ✓ | ✓ | ✓ | ✓ | ✓ | ✓* | ✓ | ✓ | ✓ | NR | NR | ✓ | NR | NR | NR | NR | NR | NR | NR | NR | NR | NR | NR | NR | NR |
| Timing of specimen collection stated | | ✓ | ✓ | ✓ | ✓ | ✓ | ✓ | ✓ | ✓ | ✓ | ✓ | ✓ | ✓* | ✓ | ✓ | ✓ | ✓ | ✓ | ✓ | ✓ | ✓ | ✓ | NR | ✓ | ✓ | ✓ |
| Duration of the study stated | | ? | ✓ | ? | ? | ✓ | ✓* | ? | ✓ | ✓* | ? | ? | ? | ? | ✓ | ? | ✓ | ? | ? | ✓ | ? | ? | ? | ? | ? | ? |
| Toxicity evaluation of the vaccination | | NR | ✓ | NR | NR | NR | NR | NR | ✓ | NR | NR | NR | NR | NR | NR | NR | NR | NR | NR | NR | NR | NR | NR | ✓ | NR | NR |
| Sample size calculation or N=5/group | | ✓ | ✓ | ✓ | ✓ | ✓ | ✓* | ✓ | ✓ | ✓* | x | x | ✓ | x | x | x | x | x | ✓* | x | x | x | ✓ | x | NR | x |
| Retrievable neutralization assay data | | ✓ | ✓ | ✓ | ✓ | x | ✓ | ✓ | ✓ | ✓ | ✓ | ✓ | ✓ | ✓ | ✓ | ✓ | ✓ | ✓ | ✓ | ✓ | ✓ | ✓ | x | ✓ | ✓ | ✓ |
| Titer/infectivity information for virus used for neutralization provided | | ✓ | ✓ | NR | ✓ | ✓ | NR | ? | ✓ | NR | ✓ | NA | NA | NR | ✓ | NR | ✓ | ✓ | ✓ | ✓ | ✓ | NR | NR | ✓* | ✓ | ✓* |
| Neutralization concentrations/dilutions provided | | ✓ | ✓ | ✓ | ✓ | ? | ✓ | ✓ | ? | ✓ | ✓ | ✓ | ? | ✓ | ✓ | ✓ | ? | ✓ | NA | NA | ✓ | NR | ✓ | ? | ✓ | ? |
| Neutralization timepoint provided | | ✓ | ✓ | ✓ | ✓ | ✓ | ✓ | ✓ | ✓ | ✓ | ✓ | ✓ | ✓ | ✓ | ✓ | ? | ✓ | ✓ | NA | NA | ✓ | NR | NR | ? | ? | ✓ |
| Statistical analysis performed | | ✓ | ✓ | ✓ | ✓ | ✓ | ✓ | NR | ✓ | ✓ | NR | NR | ✓ | ✓ | NR | NR | NR | NR | NR | NR | NR | NR | NR | NR | NR | NR |
| Statistical methods provided | | ✓ | ✓ | ✓ | ✓ | ✓ | ✓ | NR | ✓ | ✓ | NA | NR | ✓ | ✓ | NA | NA | NA | NA | NA | NA | NA | NA | NA | NA | NA | NA |
| Statement of compliance with regulatory requirements | | ✓ | ✓ | ✓ | ✓ | NR | ✓ | NR | ✓ | ✓ | NR | ✓ | ✓ | ✓ | NR | ✓ | NR | NR | NR | NR | NR | NR | NR | NR | NR | NR |
| Statement regarding possible conflict of interest | | ✓ | ✓ | ✓ | ✓ | ✓ | ✓ | NR | ✓ | NR | ✓ | ✓ | NR | ✓ | NR | NR | NR | NR | NR | NR | NR | NR | NR | NR | NR | NR |
| Publication in peer-reviewed journal | | ✓ | ✓ | ✓ | ✓ | ✓ | ✓ | ✓ | ✓ | ✓ | ✓ | ✓ | ✓ | ✓ | ✓ | ✓ | ✓ | ✓ | ✓ | ✓ | ✓ | ✓ | ✓ | ✓ | ✓ | ✓ |
| *Quality score* | | 14 | 16 | 12 | 13 | 12 | 11 | 8 | 15 | 10 | 8 | 8 | 9 | 10 | 9 | 7 | 7 | 6 | 6 | 7 | 7 | 5 | 4 | 6 | 7 | 6 |
| *Quality rating* | | Very high | Very high | High | Very high | High | High | Moderate | Very high | High | Moderate | Moderate | Moderate | High | Moderate | Moderate | Moderate | Poor | Poor | Moderate | Moderate | Poor | Poor | Poor | Moderate | Poor |
| ✓ = satisfactory  ✓* = partially satisfactory  x = unsatisfactory  NR = not reported  ? = unclear  NA = not applicable |  |  |  |  |  |  |  |  |  |  |  |  |  |  |  |  |  |  |  |  |  |  |  |  |  |  |

|  | | Emini et al., 1989 | Morgan et al., 1988a | Morgan et al., 1988b | Emini et al., 1988 | David et al., 1988 | Epstein et al., 1986 | Mackett et al., 1985 | Epstein et al., 1985 | Morgan et al., 1984 | North et al., 1982 | Thorley-Lawson et al., 1979 |
| --- | --- | --- | --- | --- | --- | --- | --- | --- | --- | --- | --- | --- |
| Clear objectives and methodology of proposed study | | ✓ | ✓ | ✓ | ✓ | ✓ | ✓ | ✓ | ✓ | ✓ | ✓ | ✓ |
| Masked assessment of outcome (analysis blinded) | | NR | NR | NR | NR | NR | NR | NR | NR | NR | NR | NR |
| Details of formulation, dosage and route of vaccine being administered | | ✓ | ✓ | ✓ | ✓ | ✓ | ✓ | ✓ | x | ✓ | ✓ | ✓ |
| Negative immunization controls included | | ✓ | ✓ | ✓ | x | x | ✓ | ✓ | ✓* | x | x | ✓ |
| Positive immunization controls included | | NR | NR | NR | ✓ | x | x | x | x | ✓* | x | NR |
| Timing of specimen collection stated | | ✓ | ✓ | NR | ✓ | ✓ | x | ✓ | NR | ✓ | ✓ | ✓ |
| Duration of the study stated | | ? | ? | ? | ? | ? | ? | ? | ? | ? | ? | ? |
| Toxicity evaluation of the vaccination | | NR | NR | NR | NR | NR | NR | NR | NR | NR | NR | NR |
| Sample size calculation or N=5/group | | x | x | x | x | x | x | x | x | x | x | x |
| Retrievable neutralization assay data | | ✓ | x | x | ✓ | x | ✓ | ✓ | ✓ | x | x | ✓ |
| Titer/infectivity information for virus used for neutralization provided | | ✓* | ✓* | ✓* | NR | NR | ✓* | NR | ✓* | ✓ | NR | NR |
| Neutralization concentrations/dilutions provided | | ✓ | NR | NR | ✓ | NR | NR | NR | NR | ✓ | ✓ | ✓ |
| Neutralization timepoint provided | | ✓ | NR | NR | ✓ | NR | ✓ | ✓ | NR | ✓ | ✓ | ✓ |
| Statistical analysis performed | | NR | NR | NR | NR | NR | NR | NR | NR | NR | NR | NR |
| Statistical methods provided | | NA | NA | NA | NA | NA | NA | NA | NA | NA | NA | NA |
| Statement of compliance with regulatory requirements | | NR | NR | NR | NR | NR | NR | NR | NR | NR | NR | NR |
| Statement regarding possible conflict of interest | | NR | NR | NR | NR | NR | NR | NR | NR | NR | NR | NR |
| Publication in peer-reviewed journal | | ✓ | ✓ | ✓ | ✓ | ✓ | ✓ | ✓ | ✓ | ✓ | ✓ | ✓ |
| *Quality score* | | 7 | 4 | 3 | 7 | 3 | 5 | 6 | 2 | 6 | 5 | 7 |
| *Quality rating* | | Moderate | Poor | Very poor | Moderate | Very poor | Poor | Poor | Very poor | Poor | Poor | Moderate |
| ✓ = satisfactory  ✓* = partially satisfactory  x = unsatisfactory  NR = not reported  ? = unclear  NA = not applicable |  |  |  |  |  |  |  |  |  |  |  |  |
